# BubR1 Insufficiency Drives Transcriptomic Alterations and Pathology Associated With Cardiac Aging and Heart Failure

**DOI:** 10.1111/acel.70160

**Published:** 2025-07-03

**Authors:** Renju Pun, Aliya L. Haas, Aradhana Thapa, Sylar R. Takafuji, Rexton M. Suzuki, Gabrielle F. Kay, Li Zheng, Michelle Waknitz, Michael H. Kim, Darren J. Baker, Jan M. van Deursen, Paul L. Sorgen, Rebekah L. Gundry, Brian J. North

**Affiliations:** ^1^ Department of Biomedical Sciences, School of Medicine Creighton University Omaha Nebraska USA; ^2^ Department of Biochemistry and Molecular Biology University of Nebraska Medical Center Omaha Nebraska USA; ^3^ Division of Cardiovascular Medicine University of Nebraska Medical Center Omaha Nebraska USA; ^4^ CHI Health Heart Institute, School of Medicine Creighton University Omaha Nebraska USA; ^5^ Department of Biochemistry and Molecular Biology Mayo Clinic Rochester Minnesota USA

**Keywords:** aging, BubR1, cardiomyopathy, heart failure, hypertrophy

## Abstract

Aging is a prominent risk factor for heart disease, driving pathological cardiac changes such as hypertrophy, fibrosis, and cellular senescence. While BubR1 has been linked to systemic aging in mammalian models, its specific role in regulating cardiac aging remains unclear. Here, we investigated how BubR1 regulates heart aging and its potential contribution to the pathogenesis of cardiac disease, including heart failure. BubR1 insufficiency in mice resulted in marked cardiac hypertrophy, increased fibrosis, and elevated markers of cellular senescence. Transcriptomic profiling revealed widespread disruption in key pathways involved in cardiac function, including ion channel regulation, cytoskeletal organization, and contractile fiber dynamics. Comparative analysis with aged hearts demonstrated shared dysregulated gene networks, linking BubR1 deficiency to age‐related cardiac dysfunction. Additionally, BubR1 hypomorphic hearts mirrored transcriptomic changes observed in end‐stage heart failure patients, and BubR1 protein levels were found to decline with age in the heart and were also significantly reduced in rodent models of heart failure and in heart failure patients. BubR1 reduction in cardiomyocytes in vitro led to an increased expression of markers of heart failure, hypertrophy, and cytoskeletal remodeling, underscoring an essential and direct role of BubR1 in maintaining cardiomyocyte health. Overall, our data suggest that BubR1 deficiency is a feature of cardiac aging and disease in humans, and that sustaining BubR1 expression may offer a potential therapeutic strategy to mitigate age‐associated cardiac decline and improve heart health in the elderly.

## Graphical abstract

Transcriptomic and cellular analyses of BubR1 hypomorphic hearts reveal disruptions in key pathways and upregulation of markers linked to impaired cardiac structure and function. Comparative transcriptomic analysis shows that BubR1 insufficiency mirrors changes seen in aged and failing hearts. A key finding of this study is that BubR1 levels decline in both aging and diseased hearts, potentially driving hypertrophy, fibrosis, senescence, and cytoskeletal remodeling, suggesting that maintaining BubR1 levels could be a therapeutic strategy to mitigate age‐related cardiac decline.

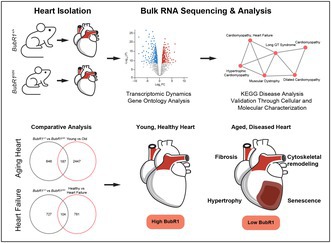

## 1 Introduction

Cardiovascular diseases (CVDs), including heart failure, remain the leading cause of mortality globally, accounting for an estimated 17.9 million deaths annually (Luo et al. 2024). The socioeconomic burden of CVDs is projected to increase significantly, with costs anticipated to rise from $393 billion in 2020 to $1.49 trillion by 2050 (Kazi et al. 2024). Aging is a well‐established risk factor for CVDs (North and Sinclair 2012). According to the American Heart Association, the prevalence of CVDs in the US is approximately 40% among individuals aged 40–59 years, escalating to 86% in those over 80 (Rodgers et al. 2019). This marked increase in CVD incidence in the elderly underscores the profound impact of age‐associated pathophysiological changes in the heart, such as hypertrophy, fibrosis, cellular senescence, and dilation, which collectively contribute to functional deterioration and heart failure (Dai et al. 2012). As such, identifying the molecular drivers underlying these age‐related changes is essential for devising therapeutic strategies aimed at preserving cardiac health.

BubR1 (budding uninhibited by benzimidazole‐related 1), encoded by the *BUB1B* gene, is a crucial component of the spindle assembly checkpoint (SAC) complex, which ensures proper chromatid segregation during mitosis (Banerjee et al. 2022). Mutations in BubR1 have been associated with mosaic variegated aneuploidy syndrome, cancer, and premature chromatid separation (Pun et al. 2024). In addition to its canonical role in mitosis, BubR1 has been implicated in non‐mitotic functions, such as regulating ciliogenesis by controlling primary cilium formation (Miyamoto et al. 2011). BubR1 insufficiency has been linked to accelerated aging phenotypes, as demonstrated in BubR1 hypomorphic mice, which express approximately 10% of wild‐type BubR1 levels (Baker et al. 2004). These mice exhibit a spectrum of aging‐related phenotypes, including reduced lifespan, cachectic dwarfism, cataracts, lordokyphosis, impaired wound healing, and a heightened susceptibility to sudden cardiac death (Baker et al. 2013; Baker et al. 2004; North et al. 2014). Cardiac complications in these mice are evident from abnormal electrocardiographic (ECG) profiles, including prolonged QT interval, shorter PR duration, prolonged QRS, increased R‐ and P‐wave amplitude, and J‐point depression (North et al. 2014). Echocardiographic analysis has demonstrated that BubR1 hypomorphic mice have significantly reduced left ventricular internal dimension (LVID). Moreover, BubR1 hypomorphic mice exhibit reduced cardiac stress resilience, succumbing three times faster than wild‐type controls to isoproterenol‐induced death, suggesting that BubR1 insufficiency directly impacts cardiac function (Baker et al. 2013). Collectively, these studies highlight a critical role for BubR1 in cardiac homeostasis and health. However the molecular basis for BubR1 in this capacity remains unknown.

Despite compelling evidence linking BubR1 deficiency to aging, its specific role in cardiac aging remains largely unexplored. Previous studies have shown that BubR1 levels decline with age in several tissues, including the brain and reproductive organs (Baker et al. 2004; Yang et al. 2017). However, whether BubR1 levels similarly decrease in the heart with age, and the consequences of its reduction on cardiac structure and function, remain unclear. In this study, we investigated the cellular and molecular cardiac phenotypes associated with BubR1 insufficiency in mice. We observed cardiac hypertrophy, increased fibrosis, and elevated markers of senescence in BubR1 hypomorphic hearts. Transcriptomic analysis revealed disrupted pathways critical for cardiac function, including ion channel activity and contractile fiber organization. Comparisons with aging hearts identified shared gene networks, linking BubR1 deficiency to cardiac aging. Additionally, BubR1 insufficiency mirrored transcriptomic changes observed in human heart failure. Notably, we show that cardiac BubR1 levels naturally decline with age and is further reduced in both mouse models of heart failure and human heart failure patients. These findings were further corroborated using in vitro models of BubR1 depletion in cardiomyocytes where BubR1 insufficiency led to an increased expression of markers of heart failure, hypertrophy, and cytoskeletal remodeling. Taken together, this study suggests that the age‐dependent decline of BubR1 in the heart is a driver of cardiac aging and disease, highlighting the therapeutic potential of targeting BubR1 loss to preserve heart health.

## 2 Results

### 2.1 Cardiac Function‐Related Genes Are Deregulated in BubR1 Hypomorphic Hearts

**FIGURE 1:**
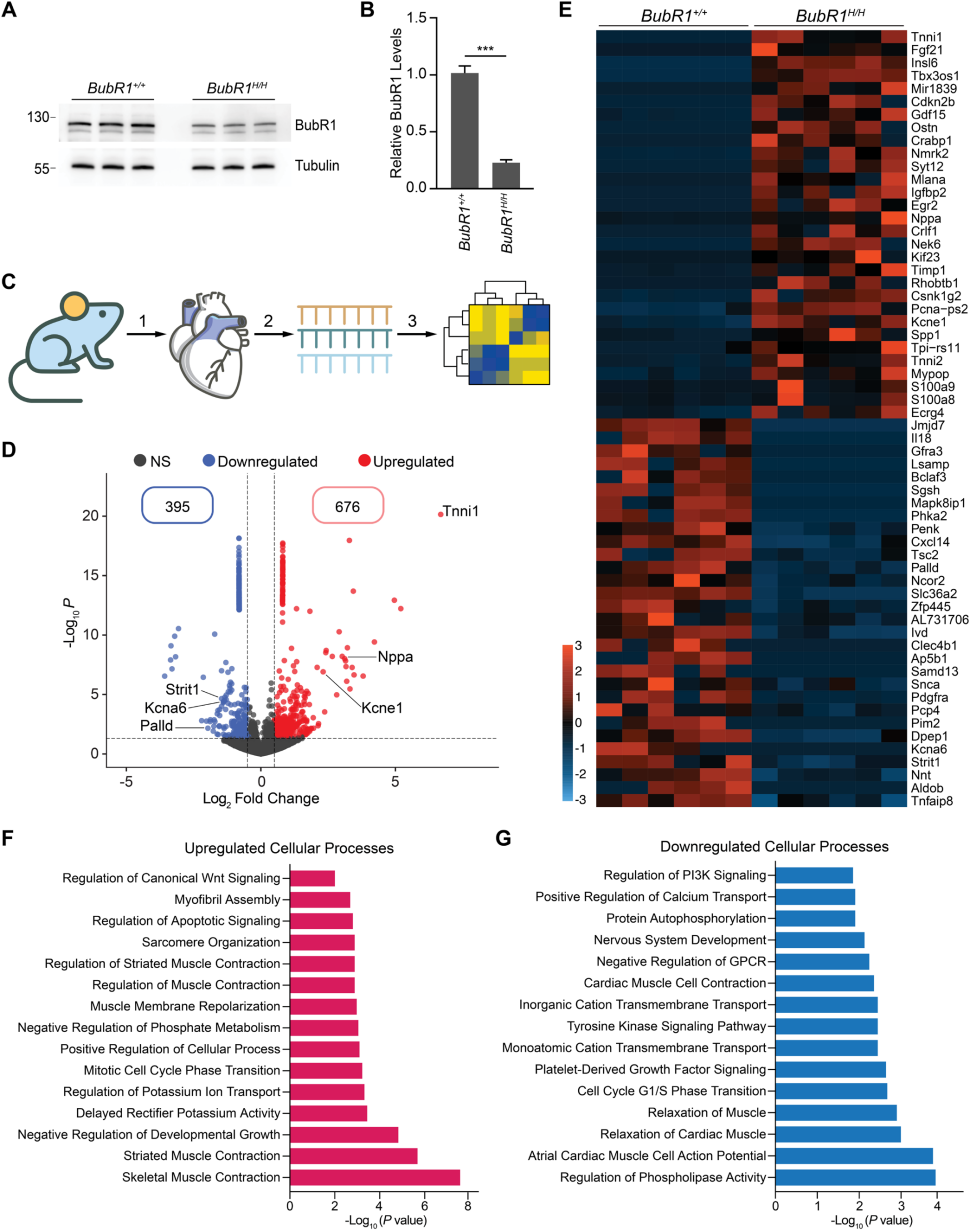
Cardiac function‐related genes are deregulated in BubR1 hypomorphic hearts. (A) Immunoblot showing BubR1 levels in hearts of 16‐week‐old wild‐type and BubR1 hypomorphic mice. (B) Quantification of BubR1 levels in A. (C) Schematic showing the workflow for bulk RNA sequencing in which 16‐week‐old mice were dissected to isolate their hearts (1) followed by tissue lysis for RNA isolation (2) and bioinformatic analysis (3). (D) Volcano plot showing the number of significantly dysregulated genes using a threshold of log_2_FC > 0.5 and *p* value < 0.05. (E) Heatmap of the top 30 upregulated and downregulated genes. (F) GO analysis carried out on biological processes of the top upregulated genes in the BubR1 hypomorphic hearts. (G) GO analysis carried out on biological processes of the top downregulated genes in the BubR1 hypomorphic hearts. Statistical significance was calculated by Student's *t*‐test. Error bars represent mean ± SEM. ***p < 0.001.

BubR1 hypomorphic mice exhibit several cardiac abnormalities, including prolonged QT syndrome and reduced tolerance to cardiac stress (Baker et al. 2013; North et al. 2014). To elucidate the global transcriptomic alterations underlying these cardiac phenotypes, we performed transcriptomic profiling of whole hearts from 16‐week‐old wild‐type and BubR1 hypomorphic mice. Immunoblotting confirmed that BubR1 hypomorphic hearts expressed reduced levels of the BubR1 protein compared to wild‐type hearts (Figure 1A,B). Total RNA was extracted from these tissues, and bulk RNA sequencing was performed, followed by comprehensive bioinformatic analysis (Figure 1C). Differential expression analysis revealed 676 genes to be upregulated and 395 genes to be downregulated in BubR1 hypomorphic hearts (Figure 1D). Notably, genes related to cardiac function, such as *Tnni1* (a cardiac troponin), *Kcne1* (a regulator of voltage‐gated potassium ion channel activity), and *Nppa* (a natriuretic hormone), were significantly upregulated (Figure 1E, Figure S1A–C). Conversely, genes like *Palld* (a cytoskeletal component), *Strit1* (a sarcoplasmic ATPase regulator), and *Kcna6* (a voltage‐gated potassium channel) were significantly downregulated in the BubR1 hypomorphic hearts (Figure 1E, Figure S1D–F). The dysregulation of these genes is associated with cardiac pathologies such as dilated cardiomyopathy, cardiac hypertrophy, and heart failure (Horsthuis et al. 2008; Watanabe et al. 2007). Upregulation of *Kcne1* leads to a prolonged QTc interval in patients with chronic heart failure (Watanabe et al. 2007). Gene Ontology (GO) analysis of upregulated genes indicated enrichment in processes such as striated muscle cell contraction, sarcomere organization, and potassium ion transport regulation (Figure 1F). In contrast, downregulated genes were involved in processes related to cardiac muscle relaxation, contraction, and regulation of atrial muscle cell action potentials (Figure 1G). These findings suggest that BubR1 insufficiency results in widespread transcriptional changes that disrupt key cardiac functions, many of which align with cardiac abnormalities in the BubR1 hypomorphic mouse model (Baker et al. 2013; North et al. 2014).

To explore sex‐specific transcriptomic alterations in BubR1 hypomorphic mice, we conducted separate bulk RNA sequencing analyses on male and female hearts. In male BubR1 hypomorphic hearts, a substantial number of genes were differentially expressed, with 820 upregulated and 642 downregulated (Figure S2A,B). GO enrichment analysis of the upregulated genes indicated dysregulation of pathways related to cytoskeletal organization and microfibril assembly, whereas the downregulated genes were predominantly associated with mitochondrial structure and function (Figure S2C,D). KEGG pathway analysis further linked these dysregulated genes to cardiovascular conditions such as dilated cardiomyopathy, long QT syndrome, and hypertrophic cardiomyopathy (Figure S2E). In contrast, female BubR1 hypomorphic hearts displayed fewer differentially expressed genes, with 189 upregulated and 204 downregulated (Figure S3A,B). GO analysis revealed enrichment in pathways related to titin and tropomyosin binding, while the downregulated genes were involved in actin cytoskeleton organization and extracellular matrix processes, particularly those involving collagen (Figure S3C,D). KEGG pathway analysis similarly identified dysregulation in pathways associated with dilated cardiomyopathy and long QT syndrome (Figure S3E).

A comparative analysis of the dysregulated genes in both sexes identified shared genes, including *Tnni1*, *Nppa*, *Gdf15*, *Cdkn1a*, and *Kcne1* (Figure S4A). GO analysis of the commonly upregulated genes indicated processes such as increased susceptibility to induced mortality, abnormal response to cardiac infarction, and enlarged myocardial infarct size (Figure S4B). Downregulated processes included abnormal fetal cardiomyocyte proliferation, dilated heart ventricle, and abnormal Purkinje cell morphology (Figure S4C). Overall, both male and female BubR1 hypomorphic hearts displayed similar dysregulation of genes critical for the maintenance of cardiac structure and function.

### 2.2 Low BubR1 Levels Induce Cardiac Hypertrophy In Vivo

**FIGURE 2:**
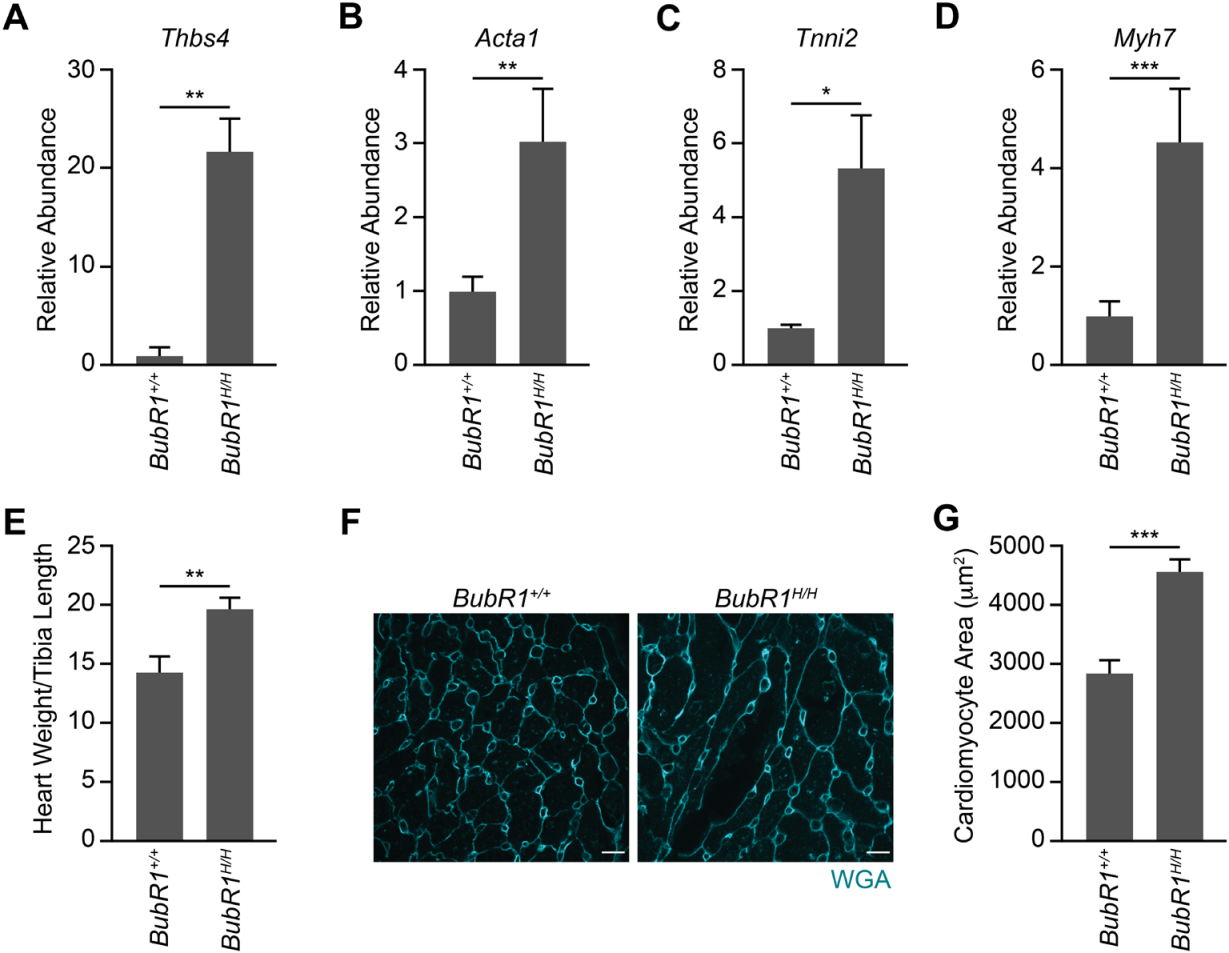
Low BubR1 levels induce cardiac hypertrophy in vivo. (A–D) Gene expression (abundance) based on DESeq2 analysis normalized against wild‐type samples of cardiac hypertrophy markers *Thbs4* (A), *Acta1* (B), *Tnni2* (C), and *Myh7* (D). (E) Heart weight to tibia length ratio of wild‐type and BubR1 hypomorphic mice (*n* = 6). (F) Confocal representative images of WGA‐stained ventricles. Scale bar, 10 μm. (G) Quantification of cardiomyocyte area in F (*n* = 6). Statistical significance was calculated by Student's t‐test. Error bars represent mean ± SEM. *p < 0.05, **p < 0.01, ***p < 0.001.

BubR1 hypomorphic mice exhibit accelerated aging phenotypes, prompting investigation into whether accelerated aging in this mouse model also extends to the heart (Baker et al. 2004). The aging myocardium is known to undergo several characteristic changes, including hypertrophy, interstitial fibrosis, and the accumulation of senescent cells (North and Sinclair 2012). Bulk RNA sequencing revealed significant upregulation of several biomarkers associated with cardiac hypertrophy in BubR1 hypomorphic hearts, including *Thbs4*, *Acta1*, *Tnni2*, and *Myh7* (Figure 2A–D). Additionally, BubR1 hypomorphic hearts exhibited a significant increase in the heart weight‐to‐tibia length ratio, consistent with the development of cardiac hypertrophy (Figure 2E). Since BubR1 hypomorphic mice are smaller than their wild type counterparts, as reflected by their lower body weight (Figure S5A), tibia length was used for normalization. However, even when normalized to body weight, the heart weight‐to‐body weight ratio remained significantly elevated in BubR1 hypomorphic mice, further supporting the notion that BubR1 insufficiency drives cardiac hypertrophy (Figure S5B). Wheat germ agglutinin (WGA) staining further confirmed a significant increase in cardiomyocyte size in BubR1 hypomorphic hearts compared to wild‐type hearts (Figure 2F,G).

### 2.3 BubR1 Hypomorphic Hearts Exhibit Increased Cellular Senescence, SASP Factors, and DNA Damage

**FIGURE 3:**
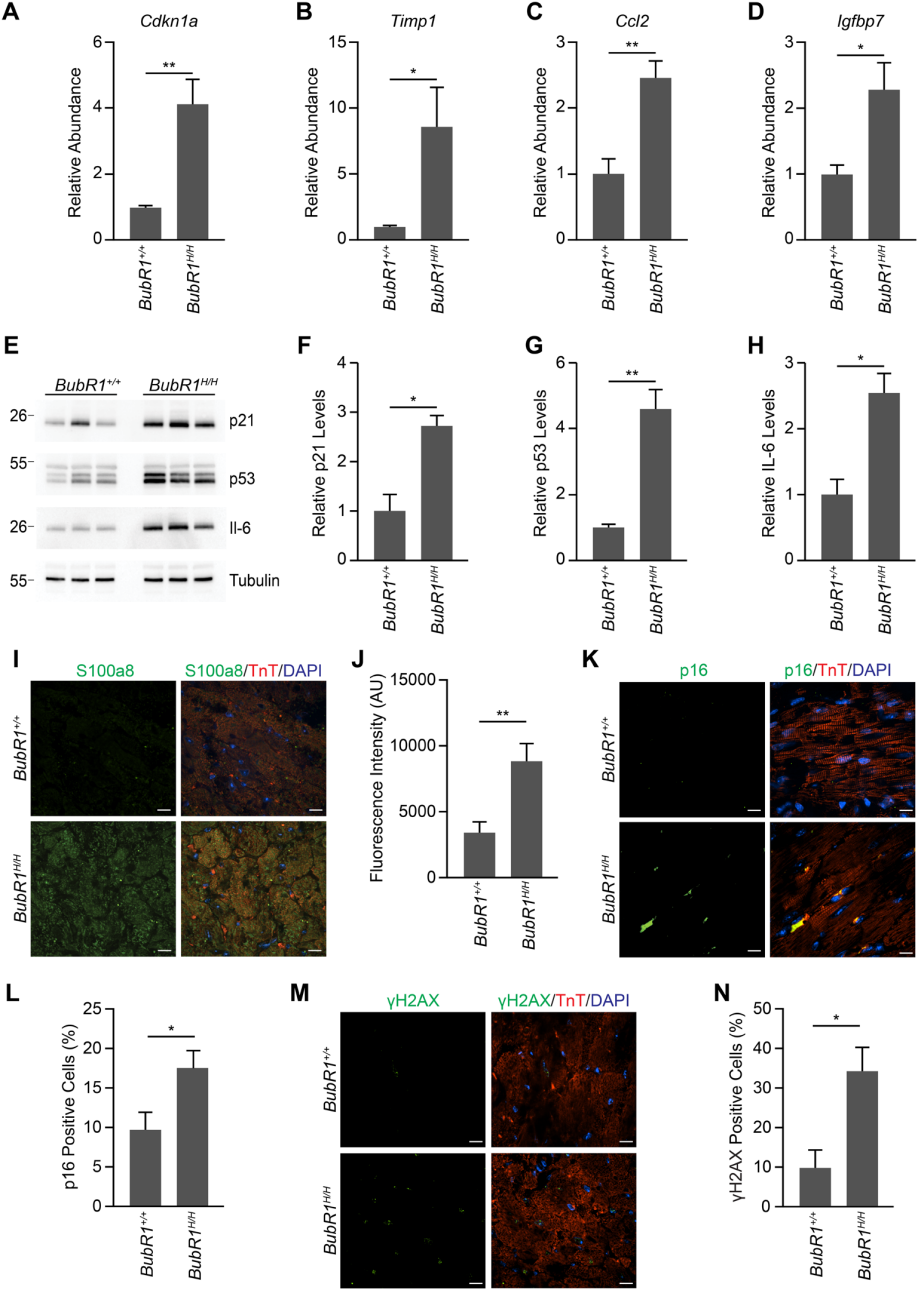
BubR1 hypomorphic hearts exhibit increased cellular senescence, SASP factors, and DNA damage. (A–D). Gene expression (abundance) based on DESeq2 analysis normalized against the wild‐type samples of senescence markers *Cdkn1a* (A), *Timp1* (B), *Ccl2* (C), and *Igfbp7* (D). (E) Immunoblot showing the protein level of senescence markers p21, p53, and IL‐6. (F–H) Quantification of relative level of p21 (F), p53 (G), and IL‐6 (H) (*n* = 3). (I) Representative confocal images of S100a8 in wild‐type and BubR1 hypomorphic hearts. Scale bar, 10 μm. (J) Quantification of S100a8 intensity shown in I (*n* = 4). (K) Representative confocal images of p16 in wild‐type and BubR1 hypomorphic hearts. Scale bar, 10 μm. (L) Percentage of p16 positive cells shown in K (*n* = 5). (M) Representative confocal images of γH2AX in wild‐type and BubR1 hypomorphic hearts. Scale bar, 10 μm. (N) Percentage of γH2AX positive cells shown in M (*n* = 5). Statistical significance was calculated by Student's *t*‐test. Error bars represent mean ± SEM. *p < 0.05, **p < 0.01.

The accumulation of senescent cells is known to contribute to cardiac remodeling, inflammation, atherosclerosis, and the progression of heart failure (Suda et al. 2023). Cyclin‐dependent kinase inhibitors, such as p21 (encoded by *Cdkn1a*), are upregulated in senescent cells and are widely recognized as hallmarks of cellular senescence (Wagner and Wagner 2022). Bulk RNA sequencing analysis revealed a significant upregulation of *Cdkn1a* in BubR1 hypomorphic hearts (Figure 3A). Moreover, senescence‐associated secretory phenotype (SASP) factors, including *Timp1* (a pro‐coagulatory factor), *Ccl2* (a chemokine ligand), and *Igfbp7* (an insulin‐like growth factor binding protein), were markedly elevated in BubR1 hypomorphic hearts (Figure 3B–D). Immunoblot analysis further corroborated these findings, showing significantly increased levels of p21 and p53 (Figure 3E). Additionally, IL‐6, a pro‐inflammatory SASP factor, was highly overexpressed in the BubR1 hypomorphic cohort, indicating enhanced senescence and SASP and its potential contribution to the observed cardiac pathology (Figure 3E–H). Bulk RNA sequencing analysis revealed that *S100A8*, a marker of inflammatory activity and a consequence of the SASP, is significantly upregulated in BubR1 hypomorphic hearts (Figure 1E). We validated this finding using immunostaining, which showed significantly higher S100A8 intensity in BubR1 hypomorphic hearts compared to wild‐type hearts (Figure 3I,J). To further assess cellular senescence, we examined the expression of p16 and p15. The number of p16‐positive cells was significantly more abundant in BubR1 hypomorphic hearts compared to wild‐type hearts (Figure 3K,L). However, the number of p15‐positive cells remained unchanged between the two groups (Figure S6A,B). Additionally, senescence‐associated β‐galactosidase (SA‐β‐gal) staining showed an increased level of senescence in BubR1 hypomorphic hearts (Figure S6C,D). Immunoblot analysis also showed increased abundance of β‐galactosidase in BubR1 hypomorphic hearts (Figure S6E,F). We also investigated whether senescence occurs specifically in the cardiomyocyte population by probing for Prominin‐2 (Prom2), a recently identified cardiomyocyte‐specific senescence marker (Maggiorani et al. 2024). Immunoblot analysis did not detect Prom2 expression in either wild‐type or BubR1 hypomorphic hearts (Figure S6E). Since cellular senescence is often triggered by elevated DNA damage, we next examined γ‐H2AX, a well‐established marker of DNA double‐strand breaks, using immunofluorescence staining. BubR1 hypomorphic hearts exhibited a significantly higher number of γ‐H2AX–positive cells compared to wild‐type controls, indicating increased DNA damage (Figure 3M–N). Given the association between telomere shortening, aging, and senescence, we also assessed telomere length in genomic DNA isolated from wild‐type and BubR1 hypomorphic hearts. No significant difference in telomere length was observed between the two groups, suggesting that the observed senescence in BubR1‐deficient hearts may not be due to telomere attrition (Figure S6G). Overall, these findings confirm the presence of aging hallmarks such as senescence and DNA damage in BubR1 hypomorphic hearts.

In addition to the SASP factors, we compared the BubR1 hypomorphic data sets with a panel of senescence‐related genes, termed SenMayo, which were identified by screening commonly regulated genes across multiple age‐related datasets (Saul et al. 2022). Male BubR1 hypomorphic mice exhibited significant upregulation of 14 SenMayo genes, whereas females displayed only nine significantly upregulated genes (Figure S7A–D). Of these, four SenMayo genes were shared between the male and female datasets. Notably, female hypomorphic mice showed a higher fold change of *Igfbp2* and *Serpine2*, while male hypomorphic mice exhibit a higher fold change of *Spp1* and *Gdf15* (Figure S7E). *Gdf15* is recognized as a SASP factor within the cardiovascular system, where its accumulation has been associated with the development of cardiac hypertrophy while *Igfbp2* is another SASP protein associated with aging (Evans et al. 2024; Wang et al. 2023). Overall, these data suggest that BubR1 hypomorphic hearts exhibit elevated levels of cellular senescence, although the specific SASP factors and the extent of senescence may vary between the sexes.

### 2.4 BubR1 Insufficiency Increases Cardiac Fibrosis Suggestive of Pathological Myocardial Remodeling

**FIGURE 4:**
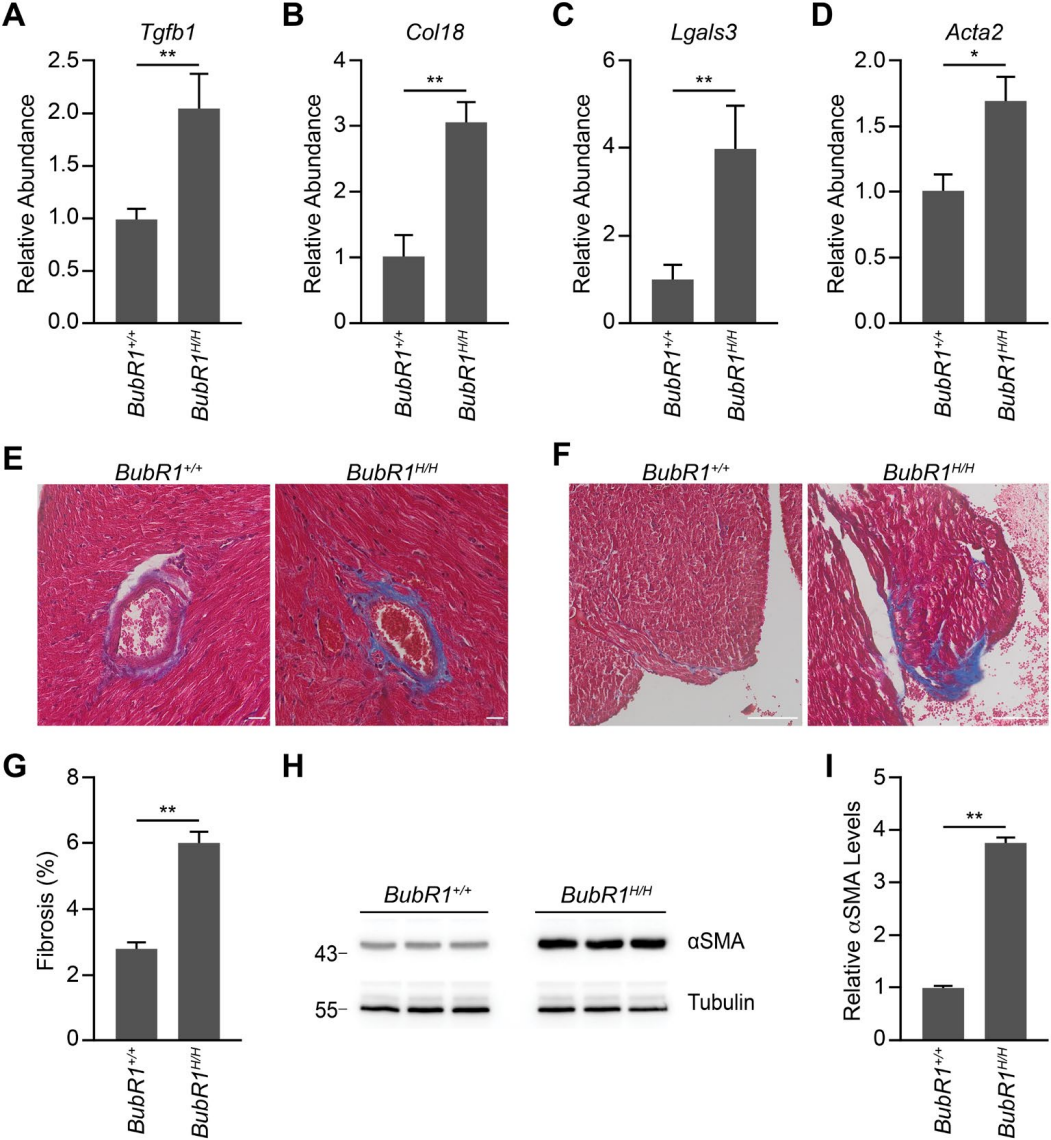
BubR1 insufficiency increases cardiac fibrosis suggestive of pathological myocardial remodeling. (A–D) Gene expression (abundance) based on DESeq2 analysis normalized against the wild‐type samples of cardiac fibrosis markers *Tgfb1* (A), *Col18* (B), *Lgals3* (C), and *Acta2* (D). (E, F) Representative images of Masson's trichrome staining in the perivascular region (E) and interstitial region (F) of wild‐type and BubR1 hypomorphic hearts. Scale bar, 20 μm and 100 μm. (G) Quantification of the percentage of fibrosis (*n* = 6). (H) Immunoblot showing protein levels of αSMA. (I) Quantification of the relative level of αSMA in H (*n* = 3). Statistical significance was calculated by Student's *t*‐test. Error bars represent mean ± SEM. *p < 0.05, **p < 0.01.

Cardiac fibrosis is characterized by the excessive deposition of extracellular matrix components in the myocardium, primarily driven by activated myofibroblasts. This pathological accumulation of fibrosis impairs chamber dilation, leading to hypertrophy and ultimately congestive heart failure (Ko et al. 2022). Bulk RNA sequencing revealed elevated expression of key fibrosis‐related genes in BubR1 hypomorphic hearts, including *Tgfb1* (Transforming Growth Factor‐β1), *Col18* (Collagen), *Lgals3* (Galectin‐3), and *Acta2* (Smooth Muscle Actin) (Figure 4A–D). Masson's trichrome staining of BubR1 hypomorphic hearts further confirmed a significant increase in fibrosis, with pronounced collagen deposition observed in both perivascular and interstitial regions (Figure 4E–G). Additionally, protein analysis revealed elevated levels of alpha smooth muscle actin (αSMA), a marker of myofibroblast activation and extracellular matrix remodeling, in BubR1 hypomorphic hearts (Figure 4H,I). These findings collectively demonstrate heightened cardiac fibrosis in BubR1 hypomorphic hearts, contributing to the observed pathological remodeling.

### 2.5 Aged Wild‐Type and Young BubR1‐Insufficient Hearts Exhibit Common Transcriptomic Changes

**FIGURE 5:**
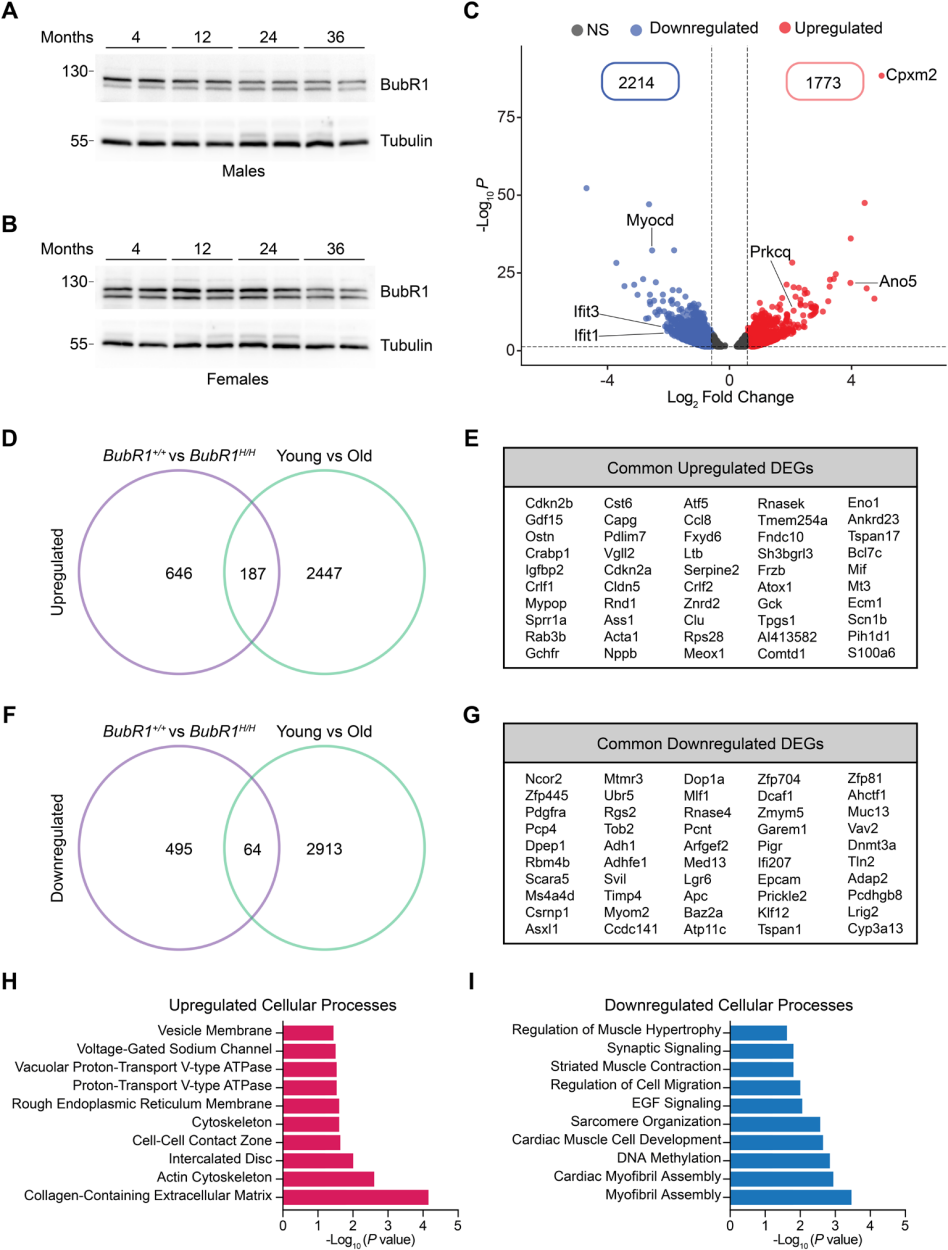
Aged wild‐type and young BubR1‐insufficient hearts exhibit common transcriptomic changes. (A) Immunoblot analysis of cardiac BubR1 levels at various ages in male mice. (B) Immunoblot image showing cardiac BubR1 levels at various ages in female mice. (C) Volcano plot showing the number of significantly dysregulated genes in 4‐month‐old versus 22‐month‐old mouse hearts using a threshold of log_2_FC > 0.5 and *p* value < 0.05. (D) Venn diagram showing the number of commonly upregulated genes between the wild‐type versus BubR1 hypomorphic dataset and the young versus aged datasets. (E) A list of top 50 commonly upregulated genes. (F) Venn diagram showing the number of commonly downregulated genes between the wild‐type versus BubR1 hypomorphic dataset and the young versus old dataset. All genes with a *p* value < 0.05 were included in the Venn diagram analysis. (G) A list of top 50 commonly downregulated genes. (H) GO analysis based on the top common upregulated genes. (I) GO analysis based on the top common downregulated genes.

Previous studies have demonstrated that BubR1 levels naturally decline with age in various organs, including the brain, testes, and ovaries (Baker et al. 2004; Yang et al. 2017). To investigate whether a similar trend occurs in the aging heart, we performed immunoblotting to assess BubR1 protein levels in male and female mice across their lifespan. In males, BubR1 expression showed a clear negative correlation with age, while in females, BubR1 levels remained stable with a decline observed at the more advanced age of 36 months (Figure 5A,B, Figure S8A,B). To further explore age‐related changes, we analyzed publicly available bulk RNA sequencing data from young (4‐month) and aged (22‐month) mice. DESeq2 analysis identified 2214 significantly downregulated and 1773 significantly upregulated genes in the aging hearts (Figure 5C). Dysregulated genes included voltage‐gated chloride ion channel genes (*Ano5*), protein kinase C isoforms (*Prkcq*), Myocardin (*Myocd*), and interferon‐related genes (*Ifit1* and *Ifit3*) (Figure S8C). GO analysis revealed that upregulated genes were enriched in pathways related to myofibrils, the sarcoplasmic reticulum, and the catenin complex, while downregulated pathways involved voltage‐gated chloride channel activity, transcription factor binding, and serine/threonine kinase activity (Figure S8D,E). A comparative analysis between wild‐type versus hypomorphic hearts and young versus aged hearts identified 187 commonly upregulated genes, including *Nppb* (natriuretic peptide), *Acta1* (skeletal muscle gene), and *Igfbp2* (insulin‐like growth factor‐binding protein) (Figure 5D,E). Notably, *Gdf15* was among the top upregulated genes shared between both datasets (Figure 5E). *Gdf15*, a member of the TGF‐beta superfamily, is highly upregulated during aging and has been strongly linked to cardiovascular diseases and senescence, making it a key marker of aging and cardiovascular mortality (Wang et al. 2023). The upregulation of *Gdf15* in BubR1 hypomorphic hearts suggests that they mimic aging‐associated changes observed in the myocardium. Regarding downregulated genes, we identified 64 genes that were commonly downregulated between the two comparative groupings. These genes included *Pcp4* (a Purkinje cell protein), *Myom2* (a titin‐binding protein), and *Adap2* (a regulator of microtubule stability) (Figure 5F,G). GO analysis revealed that the upregulated genes were associated with pathways such as the voltage‐gated sodium channel complex, cytoskeletal structures, and the collagen‐containing extracellular matrix (Figure 5H). GO analysis also showed that downregulated genes were involved in processes such as the regulation of muscle hypertrophy, regulation of striated muscle contraction, and cardiac myofibril assembly (Figure 5I). We leveraged data from the Genotype‐Tissue Expression (GTEx) project, which provides age‐associated gene sets across multiple tissues, to identify genes in BubR1 hypomorphic hearts that exhibit age‐dependent expression changes (Consortium 2015). GTEx analysis revealed that genes including *Gdf15*, *Gdf6*, *Nppa*, and *Vgll2*, are associated with cardiac aging, and were significantly upregulated in the BubR1 hypomorphic dataset (Figure S9A). Notably, *Vgll2*, which encodes a cofactor for transcriptional enhancer factor 1 (TEF‐1) and is involved in skeletal muscle development, has been identified as a robust marker of cardiovascular aging (Libiseller‐Egger et al. 2022). Furthermore, *Vgll2* is one of the shared upregulated genes between the BubR1 hypomorphic dataset and the aging cohort, suggesting its potential role in cardiac dysfunction associated with both BubR1 insufficiency and aging (Figure 5E).

Overall, these findings suggest that BubR1 hypomorphic hearts recapitulate several key molecular and cellular features of cardiac aging, including dysregulated gene expression patterns and upregulation of aging and senescence biomarkers such as *Gdf15*, indicating a potential mechanistic link between BubR1 insufficiency and accelerated cardiac aging.

### 2.6 Reduced BubR1 Levels Lead to Transcriptomic Changes Mirroring Heart Failure

**FIGURE 6:**
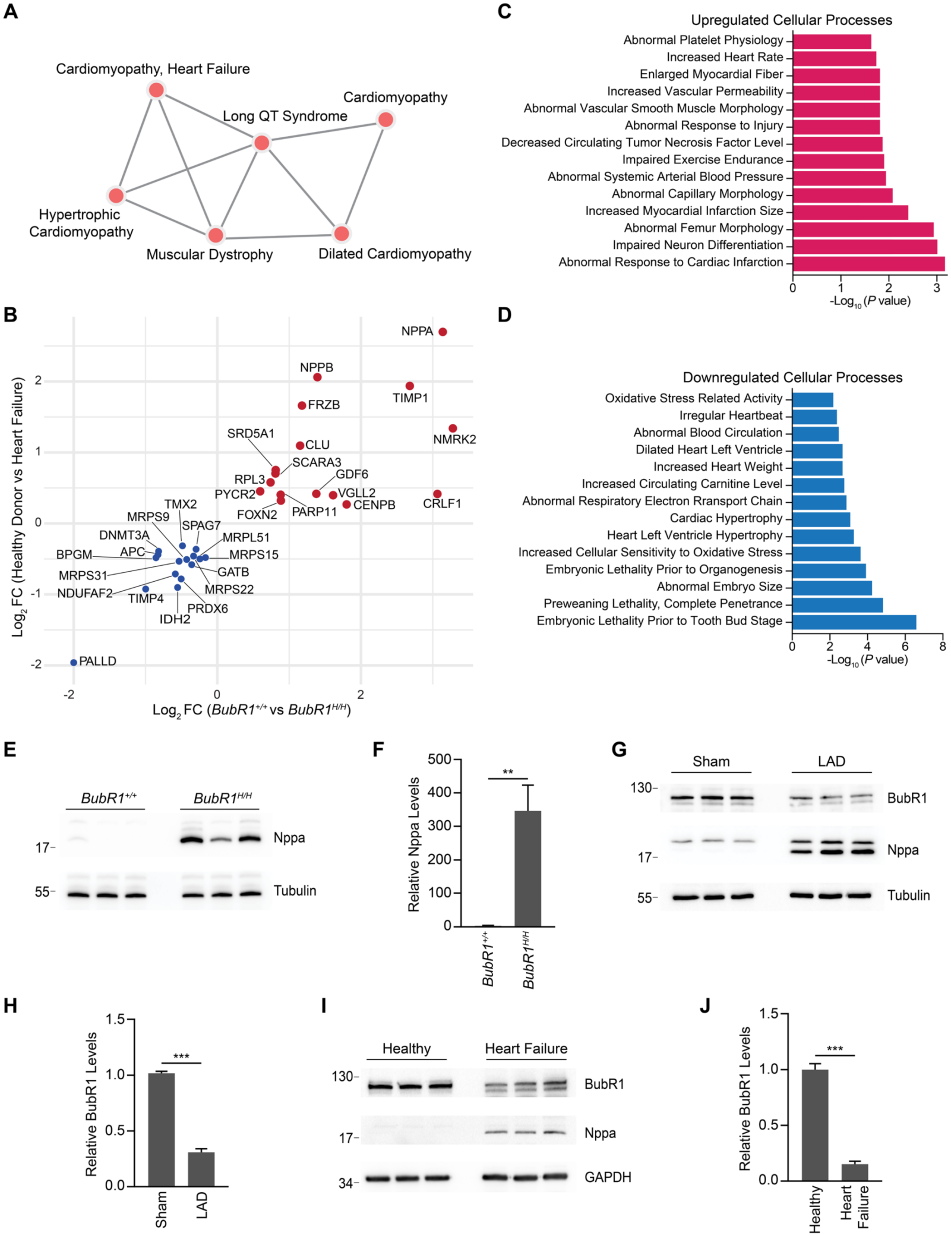
Reduced BubR1 levels lead to transcriptomic changes mirroring heart failure. (A) Disease‐based Enrichr analysis of the top differentially dysregulated genes in BubR1 hypomorphic hearts. (B) Scatterplot showing the top genes between the wild‐type versus BubR1 hypomorphic dataset and healthy donor versus heart failure dataset. (C) GO analysis carried out on biological processes of the top commonly upregulated genes. (D) GO analysis carried out on biological processes of the top commonly downregulated genes. (E) Immunoblot analysis of Nppa levels in wild‐type and BubR1 hypomorphic hearts. (F) Quantification of Nppa levels shown in E (*n* = 3). (G) Immunoblot analysis of the BubR1 levels in sham and LAD surgery mice model of heart failure. (H) Quantification of BubR1 shown in G (*n* = 3). (I) Immunoblot analysis of the BubR1 levels in healthy donor hearts and heart samples from heart failure patients. (J) Quantification of BubR1 shown in I (*n* = 3). Statistical significance was calculated by Student's *t*‐test. Error bars represent mean ± SEM. **p < 0.01, ***p < 0.001.

To determine whether BubR1 insufficiency recapitulates age‐related cardiac pathologies, we performed disease‐based KEGG analysis on the bulk RNA sequencing dataset from BubR1 hypomorphic hearts. This analysis suggested that the dysregulated genes in these hearts are associated with heart failure, dilated cardiomyopathy, hypertrophic cardiomyopathy, and long QT syndrome (Figure 6A). To further investigate the relationship between BubR1 loss and cardiac disease states, we compared transcriptomic changes observed in the BubR1 hypomorphic mice to that observed in human patients with end‐stage heart failure due to dilated cardiomyopathy. DESeq2 analysis of the heart failure cohort revealed 665 significantly downregulated and 824 significantly upregulated genes (Figure S10A). Notably, upregulated genes included natriuretic peptides (*NPPA* and *NPPB*) and Wnt family members (*CTNND2* and *WNT9A*), while downregulated genes included cardiac muscle myosin (*MYH6*), skeletal muscle associated *ACVR1*, and the cytoskeletal protein *PALLD* (Figure S10B). GO analysis of the upregulated genes in heart failure identified pathways associated with frizzled (Wnt ligand receptor) binding, hormonal activity, and receptor‐ligand interactions (Figure S10C). The activation of the Wnt signaling pathway, a hallmark of fetal gene reactivation during cardiac stress, contributes to maladaptive remodeling such as fibrosis, hypertrophy, and impaired contractility (Foulquier et al. 2018). Interestingly, the Wnt signaling pathway is also significantly upregulated in the BubR1 hypomorphic hearts (Figure 1F). Downregulated genes were associated with muscle contraction, blood circulation regulation, and voltage‐gated ion channel activity (Figure S10D). Comparative analysis between the BubR1 hypomorphic and human heart failure datasets identified 104 commonly upregulated genes and 27 commonly downregulated genes (Figure S10E,F). Among the commonly upregulated genes, *NPPA* and *NPPB* were notably elevated in both datasets (Figure 6B). *NPPA* promotes natriuresis, diuresis, and vasodilation, making its elevated levels a marker of increased cardiac stress and heart failure (Man et al. 2018). Conversely, *PALLD*, a cytoskeletal protein, was significantly downregulated in both datasets, implicating its reduction in intercalated disc dysfunction and dilated cardiomyopathy (Figure 6B). GO analysis of commonly upregulated genes revealed pathways related to heart rate regulation, enlarged myocardial fibers, and abnormal responses to cardiac infarction, while downregulated genes were associated with left ventricle dilation, left ventricular hypertrophy, and irregular heartbeat (Figure 6C,D). Immunoblotting validated the significant upregulation of Nppa in BubR1 hypomorphic hearts (Figure 6E,F). These findings indicate that BubR1 insufficiency mirrors key features of human heart failure, including upregulation of stress markers such as *NPPA*, thereby highlighting the pathological similarities between BubR1 hypomorphic hearts and advanced heart failure.

To further investigate the role of BubR1 in cardiac pathology, we assessed its expression in mouse models of heart failure. Age‐matched 16‐week‐old mice underwent either a sham surgical procedure or left anterior descending (LAD) coronary artery ligation to induce heart failure. Immunoblotting revealed elevated levels of Nppa, confirming the induction of heart failure‐associated molecular changes (Figure 6G, Figure S10G). Notably, BubR1 levels were significantly downregulated in the LAD‐ligated group, indicating that BubR1 reduction may be a component of the molecular alterations occurring during cardiac anomalies that occur during heart failure (Figure 6G,H). Next, we investigated whether a similar trend exists in human heart failure samples to assess the translational significance of BubR1 in heart disease. Interestingly, BubR1 levels were significantly reduced in the heart failure cohort compared to age‐matched donor hearts (Figure 6I,J). Concurrently, the heart failure marker Nppa was markedly elevated in the heart failure group, consistent with molecular changes associated of heart failure (Figure S10H).

We also compared senescence profiles of BubR1 hypomorphic hearts and LAD hearts. Immunoblot analysis revealed that LAD hearts exhibit elevated levels of senescence markers, including β‐galactosidase, p53, IL‐6, and p21, similar to BubR1 hypomorphic hearts (Figure S11A–F). LAD hearts also displayed a significant upregulation of Prom2, a cardiomyocyte‐specific senescence marker, suggesting that cardiomyocytes contribute to the senescence burden in the LAD model (Figure S11A,F). In contrast, BubR1 hypomorphic hearts do not show increased Prom2 expression (Figure S6E).

Heart failure is characterized by extensive remodeling of the cytoskeletal architecture, which includes an increase in the density of key cytoskeletal proteins such as desmin, tubulin, and actin (Schaper et al. 1991). These alterations impose additional mechanical load on the myocardium and restrict movement of the sarcomeres (Schaper et al. 1991). Our analysis of heart failure patient samples revealed a significant increase in tubulin levels (Figure S12A,B), consistent with findings previously reported in cardiac conditions such as hypertrophic and dilated cardiomyopathy (Schaper et al. 1991). Additionally, desmin, an intermediate filament protein predominant in skeletal muscle, was significantly elevated in the human heart failure cohort (Figure S12A,C). Notably, several faster migrating species of desmin were observed below the expected full‐length desmin molecular weight (Figure S12A), suggesting the accumulation of post‐translationally modified and truncated desmin proteins that are prone to degradation (Rainer et al. 2018). To further investigate these changes, we examined cytoskeletal protein levels in BubR1 hypomorphic hearts and found a significant increase in β‐actin and desmin while tubulin was not altered in the BubR1 hypomorphic hearts (Figure S12D–F). The elevation of β‐actin is associated with hypertrophic myocardium (Balasubramanian et al. 2010). Immunostaining for α‐actinin, a sarcomeric marker, revealed disorganized and discontinuous sarcomere structures in BubR1 hypomorphic hearts, contrasting with the well‐aligned striations in wild‐type hearts (Figure S12G,H). Overall, our data suggest that BubR1 insufficiency in mice induces remodeling and disorganization of the cytoskeletal structure, similar to changes observed in human heart failure, although specific proteins affected differ between species. However, the consistent upregulation of desmin across species, together with GO enrichment of cytoskeletal pathways, including alterations in myofibril assembly, sarcomere organization, and muscle contraction, highlights a conserved disruption of cytoskeletal architecture (Figure 1F,G, Figure S2C, and S3C,D).

### 2.7 BubR1 Depletion in AC16 Cardiomyocytes Induces Hypertrophy but Not Senescence

BubR1 hypomorphic animals exhibit multiple age‐associated phenotypes, making it challenging to determine whether the cardiac defects observed in BubR1 hypomorphic hearts are a direct consequence of BubR1 deficiency or a secondary effect of systemic aging and whole‐body functional decline. To address this, we recapitulated BubR1 deficiency in the human AC16 cardiomyocyte cell line using shRNA‐mediated BubR1 depletion. Immunoblotting analysis revealed that depleting BubR1 in AC16 cells led to a significant upregulation of Nppa (Figure S13A). Similarly, desmin expression was also elevated in BubR1 shRNA‐expressing cardiomyocytes, consistent with the upregulation of desmin observed in BubR1 hypomorphic hearts (Figure S13A). Since BubR1 hypomorphic hearts exhibit cardiac hypertrophy and we detected upregulation of key hypertrophy markers including Nppa, we assessed cellular hypertrophy using Phalloidin staining (Figure S13A). Quantification of cell surface area showed that BubR1 depleted AC16 cells had a significantly larger cell area compared to control cells (Figure S13B,C).

Next, we determined whether reducing BubR1 levels in AC16 cells induces senescence. Interestingly, the SA‐β‐gal assay showed no significant difference between control and BubR1‐depleted cells (Figure S13D,E). Similarly, senescence markers such as p21 and p53 levels remained unchanged (Figure S13F). The expression of Prom2 was minimal and not altered between control and BubR1‐depleted cells, suggesting that BubR1 reduction does not induce senescence in AC16 cardiomyocytes (Figure S13F). Overall, BubR1 insufficiency appears to drive cardiac phenotypes, including hypertrophy, elevated expression of the heart failure marker Nppa, and increased cytoskeletal proteins such as desmin, which may be independent of its effects on senescence.

## 3 Discussion

Reduced expression of BubR1 in mice has been shown to accelerate aging and age‐related phenotypes, such as cachectic dwarfism, cataracts, and reduced lifespan (Baker et al. 2004). These mice also exhibit cardiac abnormalities, including arrhythmias, prolonged QT syndrome, and reduced tolerance to cardiac stress, conditions that become more prevalent with age (Baker et al. 2013; North et al. 2014). Despite these observations, the specific structural and molecular consequences of BubR1 deficiency in the heart remain unexplored. In this study, we used young adult BubR1 hypomorphic mice to uncover the structural, cellular, and transcriptomic alterations in their cardiac system and determine their potential relevance to cardiac aging and heart failure.

We found that similar to many other tissues, BubR1 naturally declines with age in the heart. Given that BubR1 insufficiency leads to multiple cardiac defects, this natural decline may contribute to the increased prevalence of cardiovascular diseases in aging populations. Interventions that stabilize BubR1 levels in the aging myocardium may offer a promising strategy to mitigate the rise in CVDs. Supporting this notion, transgenic mice that sustain BubR1 expression levels exhibit increased cardiac stress tolerance and reduced interstitial fibrosis (Baker et al. 2013). BubR1 degradation is regulated by acetylation at lysine K668 through the action of acetylase CBP (CREB‐binding protein), while the NAD^+^‐dependent deacetylase SIRT2 targets BubR1 for deacetylation to promote its stabilization (North et al. 2014). Treatment with the NAD^+^ precursor NMN (nicotinamide mononucleotide) reverses the age‐dependent decline of BubR1 (North et al. 2014). Therefore, NMN supplementation could be a method of stabilizing BubR1 in the aging heart. Interestingly, our analysis of BubR1 levels across the lifespan revealed that BubR1 is more stable in female hearts compared to male hearts. If this sex‐dependence in rate of BubR1 decline is also present in human hearts, this observation aligns with the fact that heart failure prevalence is higher in males aged 45–64 compared to females, while the prevalence becomes similar in both sexes after age 75 (Mehta and Cowie 2006). Thus, this rapid decline of BubR1 in males may contribute to the higher incidence of heart failure during middle age. Interestingly, male BubR1 hypomorphic mice were also more responsive to enhanced survival mediated by ectopic SIRT2 expression (North et al. 2014). In summary, the natural age‐related decline of BubR1 in the heart warrants further investigation to elucidate its full impact on cardiac health.

Cellular and tissue analysis demonstrated that BubR1 hypomorphic hearts suffer from cardiac hypertrophy, fibrosis, and heightened senescence, which accumulates when cells age, suggesting that cardiac phenotypes in a BubR1 deficient heart could be a result of accelerated aging. One hallmark of aging is the reactivation of the fetal gene signature (Foulquier et al. 2018). During cardiac remodeling in response to heart failure, the activation of fetal genes represents a pathological attempt by the heart to cope with increased stress and damage (Dirkx et al. 2013). Despite initial protective intents, the persistent expression of fetal genes in the failing heart contributes to detrimental changes in myocardial structure and function (Dirkx et al. 2013). Our differential gene expression analysis revealed that genes associated with the Wnt signaling pathway are upregulated in both BubR1 hypomorphic and human heart failure datasets. Since Wnt signaling regulates cardiomyocyte proliferation during cardiac development, its reactivation is a critical part of the fetal gene reactivation process (Edwards et al. 2020; Foulquier et al. 2018). BubR1 and its binding partner Mad2 have been implicated in insulin signaling through their role in insulin receptor endocytosis (Choi et al. 2016). The upregulation of Wnt‐related genes observed in BubR1 hypomorphic hearts may be a result of changes in insulin signaling (Tian et al. 2021), a connection that could be explored in future studies. Similarly, the heightened expression of Nppa is also part of the fetal gene reactivation program since Nppa is expressed in the developing embryonic heart but is strongly reduced in the ventricle post‐birth (Houweling et al. 2005). However, Nppa expression is highly upregulated in the adult heart undergoing cardiac stress such as during heart failure (Horsthuis et al. 2008). Our comparative analysis showed that Nppa was the topmost upregulated common gene in both the BubR1 hypomorphic and the human heart failure datasets. Overall, BubR1 insufficiency leads to transcriptomic changes akin to heart failure. Given it has been postulated that the BubR1 hypomorphic mice die in a manner reminiscent of sudden cardiac death due to cardiac conduction defects (Baker et al. 2013; North et al. 2014), our results based on transcriptomic profiling suggest that a reduction in BubR1 levels may establish a pre‐heart failure state.

Our analysis of BubR1 in the LAD heart failure model revealed that BubR1 protein levels decline with heart failure induction. Similarly, human heart failure samples exhibited markedly reduced BubR1 levels compared to healthy donor hearts, suggesting that BubR1 downregulation may be a critical component of heart failure pathology. To further investigate this, we depleted BubR1 in human cardiomyocytes and observed increased expression of desmin and Nppa. Furthermore, BubR1‐depleted cardiomyocytes exhibited a significant increase in cell surface area, reinforcing the role of BubR1 in regulating cardiomyocyte hypertrophy. Cytoskeletal remodeling, including microtubule densification and desmin aggregation, is a known feature of heart failure that disrupts sarcomere function, leading to reduced contractility and an increased likelihood of heart failure (Rainer et al. 2018; Schaper et al. 1991). Moreover, our bulk RNA sequencing data suggest that processes related to cytoskeletal architecture are disrupted in BubR1 hypomorphic hearts, further supporting the role of BubR1 in cytoskeletal remodeling. Despite these changes, BubR1 reduction on its own did not elevate senescence in vitro in AC16 cardiomyocytes. This observation is further supported by the observation that BubR1 hypomorphic mice do not express Prom2, a cardiomyocyte‐specific senescence marker, implying that the cardiac defects observed in BubR1 hypomorphic hearts are not likely to be secondary effects of heightened cardiomyocyte senescence. This stands in contrast to the LAD‐induced myocardial infarction model, in which BubR1 downregulation coincides with Prom2 upregulation, indicative of cardiomyocyte senescence. This suggests that the senescent cell burden in BubR1 hypomorphic hearts may arise from non‐cardiomyocyte populations, such as endothelial or fibroblast cells. Another plausible explanation could be that BubR1 downregulation alone is not sufficient to trigger senescence under baseline conditions but rather sensitizes cardiomyocytes to undergo senescence in response to additional stressors, such as ischemic injury induced by LAD ligation. Further studies will be needed to test this hypothesis.

While the most well characterized function of BubR1 is to regulate mitotic progression through controlling the SAC, our data suggest additional non‐mitotic roles for BubR1. Further supporting the idea that SAC proteins such as BubR1 have non‐mitotic functions in post‐mitotic tissues, a recent study demonstrated that SAC proteins suppress dendritic microtubule dynamics in post‐mitotic neurons (Hertzler et al. 2020). This finding highlights that SAC proteins can serve structural and regulatory roles in fully differentiated, non‐dividing cells, which is particularly relevant in the context of cardiomyocytes. Given that cytoskeletal integrity is crucial for cardiac function, these findings provide further rationale for exploring the role of BubR1 beyond mitosis in the adult heart. Future studies should investigate interventions targeting cytoskeletal proteins in BubR1‐deficient hearts to elucidate the role of this mechanism in cardiac function and pathology.

While we focused our analysis on transcriptomic changes in the hearts of BubR1 hypomorphic mice, one limitation of this study is that functional consequences in parallel cohorts were not assessed. Previous studies have demonstrated that BubR1 hypomorphic mice display cardiac dysfunction, including ECG abnormalities such as a prolonged QT interval (North et al. 2014), whose prevalence increases with age (Guettler et al. 2019; Reardon and Malik 1996). However, we did not examine whether this specific cohort of BubR1 hypomorphic animals displayed similar ECG abnormalities as observed previously (North et al. 2014), although they are on the same genetic background. Nonetheless, our transcriptomic analysis revealed enrichment of disease signatures not only for heart failure, but also for long QT syndrome in BubR1 hypomorphic hearts. Notably, *Kcne1*, a gene associated with long QT syndrome which is involved in the regulation of cardiac repolarization, was significantly upregulated in BubR1 hypomorphic hearts (Watanabe et al. 2007) (Figure 1D,E). This supports a mechanistic link between BubR1 deficiency and altered electrophysiological properties of the heart. Future studies incorporating functional longitudinal analysis by echocardiography and electrophysiological assessments in BubR1 hypomorphic hearts, or conditional BubR1 knockouts that lack BubR1 in the adult heart or within specific subsets of cardiomyocytes, would be useful to further validate these associations and determine the extent to which the transcriptomic changes reflect relevant pathophysiological phenotypes seen in the heart in response to aging and heart failure.

Collectively, our study positions BubR1 as a potential key player in maintaining adult cardiac structure and function. Therefore, its decline may serve as both a biomarker and a potential therapeutic target for heart anomalies, particularly those exacerbated by aging.

## 4 Methods

### 4.1 Animals

We utilized 16‐week‐old mice of both sexes in all our studies. All mice were bred and maintained on a C57BL/6J (Jackson Laboratories) background at the AAALAC‐accredited Creighton University animal resource facility. Experiments using BubR1 hypomorphic mice were carried out under the authority of and approval by the Creighton University Institutional Animal Care and Use Committee Policies and Procedures. LAD and sham surgery were carried out at UNMC under the approval of the UNMC Institutional Animal Care and Utilization Committee.

### 4.2 RNA Isolation and Bulk RNA Sequencing

Total RNA from hearts was isolated using the RNeasy Fibrous Tissue Kit (Qiagen, 74704). Briefly, 10 mg of heart tissue was cut from the apex and lysed following the manufacturer's protocol. RNA integrity (RIN) was assessed using the Agilent TapeStation 4200, and samples with a RIN value > 8.0 were sequenced with Illumina Hiseq 4000 through LC Sciences. Quality control of the raw sequencing data was performed using FastQC. The fastq reads were aligned to the mouse reference genome GRCm38/mm10 using HiSat2 and quantified with StringTie. Subsequent analysis was performed in RStudio (version 4.4.0) using Bioconductor and the DESeq2 package. Differential gene expression analysis identified significantly dysregulated genes, which were visualized with heatmaps and volcano plots using the “pheatmap” and “EnhancedVolcano” packages, respectively. Gene abundance values were calculated using DESeq2. To predict biological functions and diseases based on the differentially expressed genes, we used an enrichment analysis tool, Enrichr, to conduct GO and KEGG analysis (Chen et al. 2013).

### 4.3 Coronary Artery Ligation (LAD Surgery)

Induction of heart failure through myocardial infarction was performed as previously described (Gao et al. 2010). Hearts from both sham and LAD groups were collected 8 weeks post‐surgery for analysis. Briefly, mice were anesthetized with 2% isoflurane and a 1.2 cm incision was made in the skin over the left chest. A small hole was made on the fourth intercostal space providing access to the heart. The left coronary artery was located, sutured, and ligated with a 6–0 silk suture. The heart was repositioned within the thoracic cavity and the open wound was closed with suture. Post operative recovery was quick and occurred within 5 min of surgery. Sham surgery underwent the same procedure without the occlusion of the left coronary artery.

### 4.4 Heart Failure Patient Samples

Samples were obtained in compliance with institutional review board approvals and collected as deidentified samples through the Nebraska Cardiovascular Biobank and Registry at the University of Nebraska Medical Center (PRO643‐17‐EP). Samples collected during rapid autopsy (non‐failing) or at time of heart transplant (failing) were placed in cardioplegia solution on ice and transported to the research laboratory for processing. Tissues were dissected and cryopreserved in a solution of 10% dimethyl sulfoxide, 10% fetal bovine serum, 80% Dulbecco's Modified Eagle Medium, and stored in liquid nitrogen until use (Luecke et al. 2023; Wojtkiewicz et al. 2022). Further patient details are provided in Table S4.

### 4.5 Statistics

All graphs represent +/− SEM. The difference between two means were analyzed using two‐tailed unpaired Student's *t*‐test in GraphPad Prism version 10. A *p* value < 0.05 were deemed to be statistically significant. Statistical analysis of differential gene expression in the bulk RNA sequencing data was performed using the DESeq2 package. The significance threshold for the identification of differentially expressed genes was set as *p* < 0.05.

## Author Contributions

Renju Pun, Michael H. Kim, and Brian J. North: conceptualization. Renju Pun, Aliya L. Haas, Aradhana Thapa, Sylar R. Takafuji, Rexton M. Suzuki, Gabrielle F. kay, Li Zheng, Michelle Waknitz, and Brian J. North: investigation. Aliya L. Haas and Renju Pun: bioinformatics. Renju Pun and Brian J. North: writing. Renju Pun, Darren J. Baker, Jan M. van Deursen, Paul L. Sorgen, Rebekah L. Gundry, and Brian J. North: review and editing. Michael H. Kim and Brian J. North: funding acquisition.

## Conflicts of Interest

Darren J. Baker and Jan M. van Deursen have a potential financial interest related to this research. Jan M. van Deursen is a cofounder of Unity Biotechnology, and both Darren J. Baker and Jan M. van Deursen are co‐inventors on patents held by Mayo Clinic, patent applications licensed to or filed by Unity Biotechnology, and a Unity Biotechnology shareholder. Research in the Baker laboratory has been reviewed by the Mayo Clinic Conflict of Interest Review Board and is being conducted in compliance with Mayo Clinic Conflict of Interest policies. The other authors have no conflicts interest to declare.

## Supporting information


Appendix S1.

**Table S1.** Significantly upregulated and downregulated genes in BubR1 hypomorphic hearts compared to wild‐type hearts (Sheet 1). Significantly upregulated and downregulated genes in male BubR1 hypomorphic hearts compared to wild‐type hearts (Sheet 2). Significantly upregulated and downregulated genes in female BubR1 hypomorphic hearts compared to wild‐type hearts (Sheet 3).

**Table S2.** Significantly upregulated and downregulated genes in young (4‐month‐old) and aged (22‐month‐old) hearts (Sheet 1). Common upregulated and downregulated genes between BubR1 hypomorphic hearts and aged mouse hearts (Sheet 2).

**Table S3.** Significantly upregulated and downregulated genes in heart failure patient samples compared to healthy donor hearts (Sheet 1). Common upregulated and downregulated genes between BubR1 hypomorphic hearts and heart failure patient samples (Sheet 2).

**Table S4.** Clinical details of the healthy donor individuals and heart failure patients.

## Data Availability

The data that support the findings of this study are openly available in NCBI Gene Expression Omnibus at https://www.ncbi.nlm.nih.gov/geo/, reference number GSE277997.
