# Supplementary material for: BubR1 Insufficiency Drives Transcriptomic Alterations and Pathology Associated With Cardiac Aging and Heart Failure
Source: Aging Cell. 2025 Jul 3;24(9):e70160. doi: 10.1111/acel.70160 (PMC12419853; doi:10.1111/acel.70160)
Supplement: Supplementary file 1 — Appendix S1. [file ACEL-24-e70160-s004.pdf]

Supplemental Material:

BubR1 Insufficiency Drives Transcriptomic Alterations and Pathology Associated with Cardiac Aging and Heart Failure

Renju Pun[1], Aliya L. Haas[1], Aradhana Thapa[1], Sylar R. Takafuji[1], Rexton M. Suzuki[1], Gabrielle F. Kay[1], Li Zheng[2], Michelle Waknitz[3], Michael H. Kim[4], Darren J. Baker[5], Jan M. van Deursen[5], Paul L. Sorgen[2], Rebekah L. Gundry[3], and Brian J. North[1,*]

Supplementary Figures and Legends:

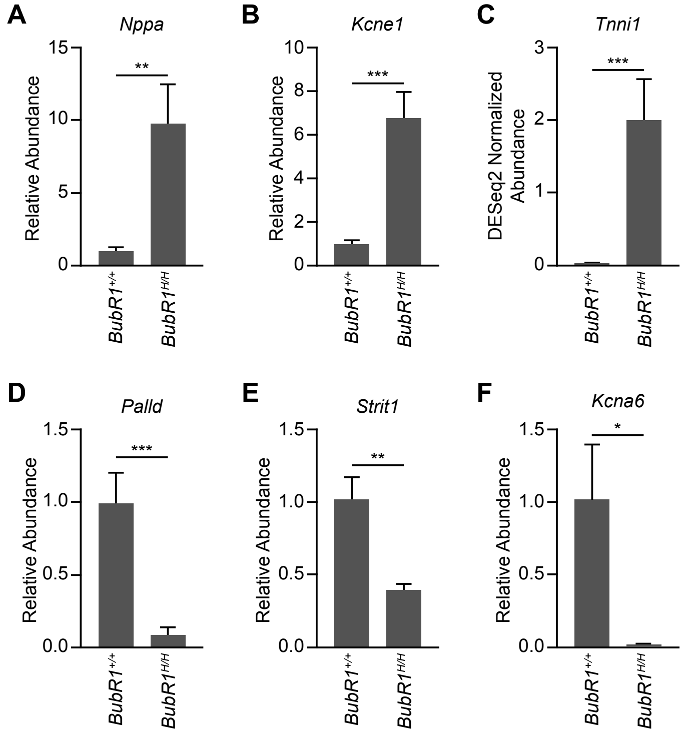

Figure S1: Dysregulation of genes related to cardiac function in BubR1 hypomorphic hearts. A-B. Gene expression (abundance) of significantly upregulated genes based on DESeq2 analysis normalized against the wild-type samples in *Nppa* (A) and *Kcne1* (B). **C.** DESeq2-normalized gene expression of *Tnni1* (expression wild-type hearts is 0 for all replicates). **D-F.** Gene expression (abundance) of significantly downregulated genes based on DESeq2 analysis normalized against the wild-type samples in *Palld* (D), *Strit1* (E), and *Kcna6* (F). Statistical significance was calculated by Student's *t*-test. Error bars represent mean ± SEM (n=6). * $p < 0.05$, ** $p < 0.01$, *** $p < 0.001$.

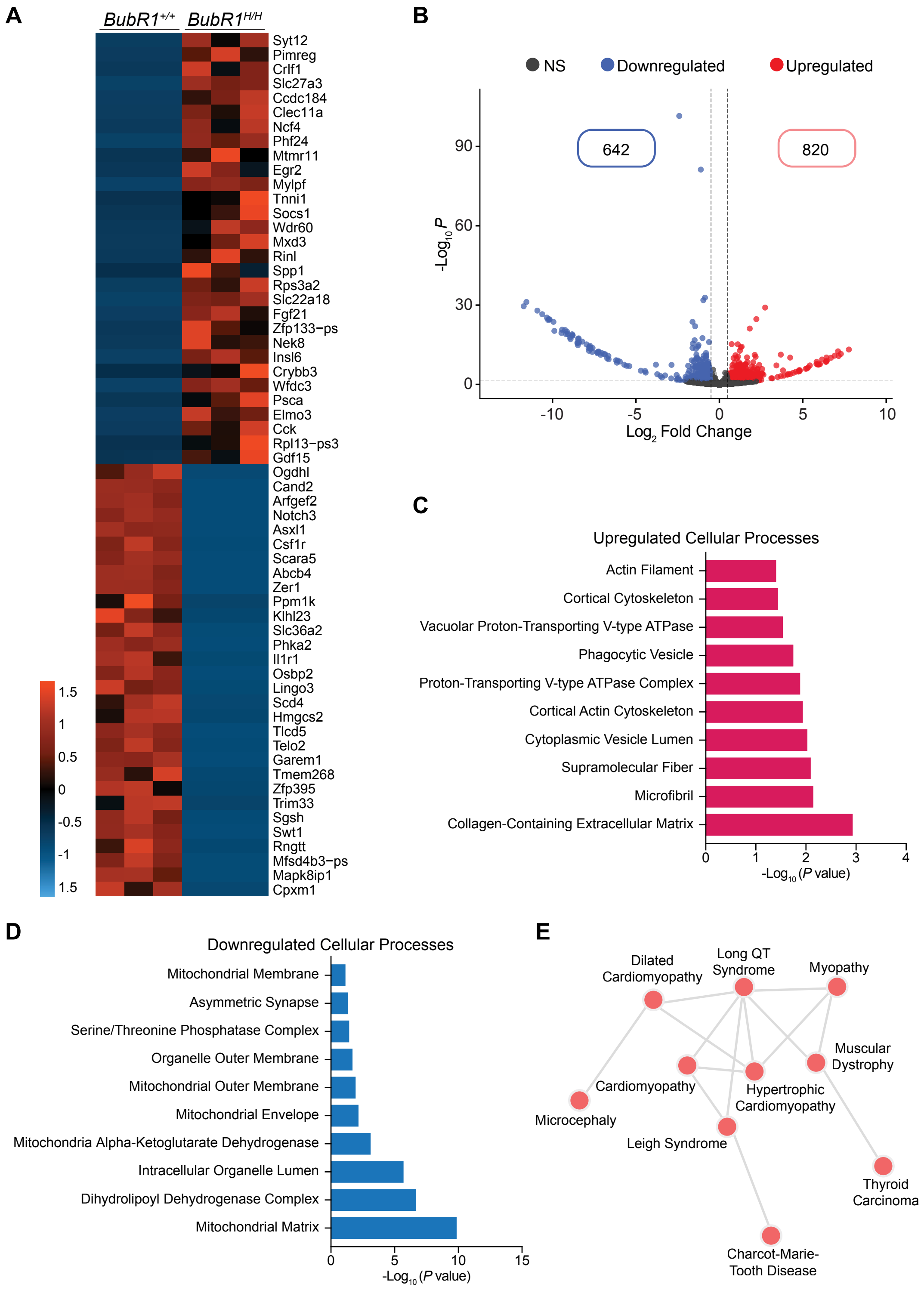
A
BubR1+/+
BubR1H/H
Syt12
Pimreg
Crlf1
Slc27a3
Ccdc184
Clec11a
Ncf4
Phf24
Mtmr11
Egr2
Mylpf
Tnni1
Socs1
Wdr60
Mxd3
Rinl
Spp1
Rps3a2
Slc22a18
Fgf21
Zfp133−ps
Nek8
Insl6
Crybb3
Wfdc3
Psca
Elmo3
Cck
Rpl13−ps3
Gdf15
Ogdhl
Cand2
Arfgef2
Notch3
Asxl1
Csf1r
Scara5
Abcb4
Zer1
Ppm1k
Klhl23
Slc36a2
Phka2
Il1r1
Osbp2
Lingo3
Scd4
Hmgcs2
Tlcd5
Telo2
Garem1
Tmem268
Zfp395
Trim33
Sgsh
Swt1
Rngtt
Mfsd4b3−ps
Mapk8ip1
Cpxm1
1.5
1
0.5
0
-0.5
-1
1.5
B
NS
Downregulated
Upregulated
642
820
90
60
30
0
-Log10 P
-10
-5
0
5
10
Log2 Fold Change
C
Upregulated Cellular Processes
Actin Filament
Cortical Cytoskeleton
Vacuolar Proton-Transporting V-type ATPase
Phagocytic Vesicle
Proton-Transporting V-type ATPase Complex
Cortical Actin Cytoskeleton
Cytoplasmic Vesicle Lumen
Supramolecular Fiber
Microfibril
Collagen-Containing Extracellular Matrix
0
1
2
3
4
-Log10 (P value)
D
Downregulated Cellular Processes
Mitochondrial Membrane
Asymmetric Synapse
Serine/Threonine Phosphatase Complex
Organelle Outer Membrane
Mitochondrial Outer Membrane
Mitochondrial Envelope
Mitochondria Alpha-Ketoglutarate Dehydrogenase
Intracellular Organelle Lumen
Dihydrolipoyl Dehydrogenase Complex
Mitochondrial Matrix
0
5
10
15
-Log10 (P value)
E
Dilated Cardiomyopathy
Long QT Syndrome
Myopathy
Muscular Dystrophy
Cardiomyopathy
Hypertrophic Cardiomyopathy
Microcephaly
Leigh Syndrome
Thyroid Carcinoma
Charcot-Marie-Tooth Disease

Figure S2: Global transcriptomic alterations in male BubR1 hypomorphic hearts. A. Heatmap of the top 30 upregulated and downregulated genes in male BubR1 hypomorphic hearts. **B.** Volcano plot showing the number of significantly dysregulated genes using a threshold of $log_2FC > 0.5$ and p value < 0.05. **C.** GO analysis carried out on biological processes of the top upregulated genes in the male BubR1 hypomorphic hearts. **D.** GO analysis carried out on biological processes of the top downregulated genes in the male BubR1 hypomorphic hearts. **E.** Diseased based Enrichr analysis on the top differentially dysregulated genes in the male BubR1 hypomorphic hearts.

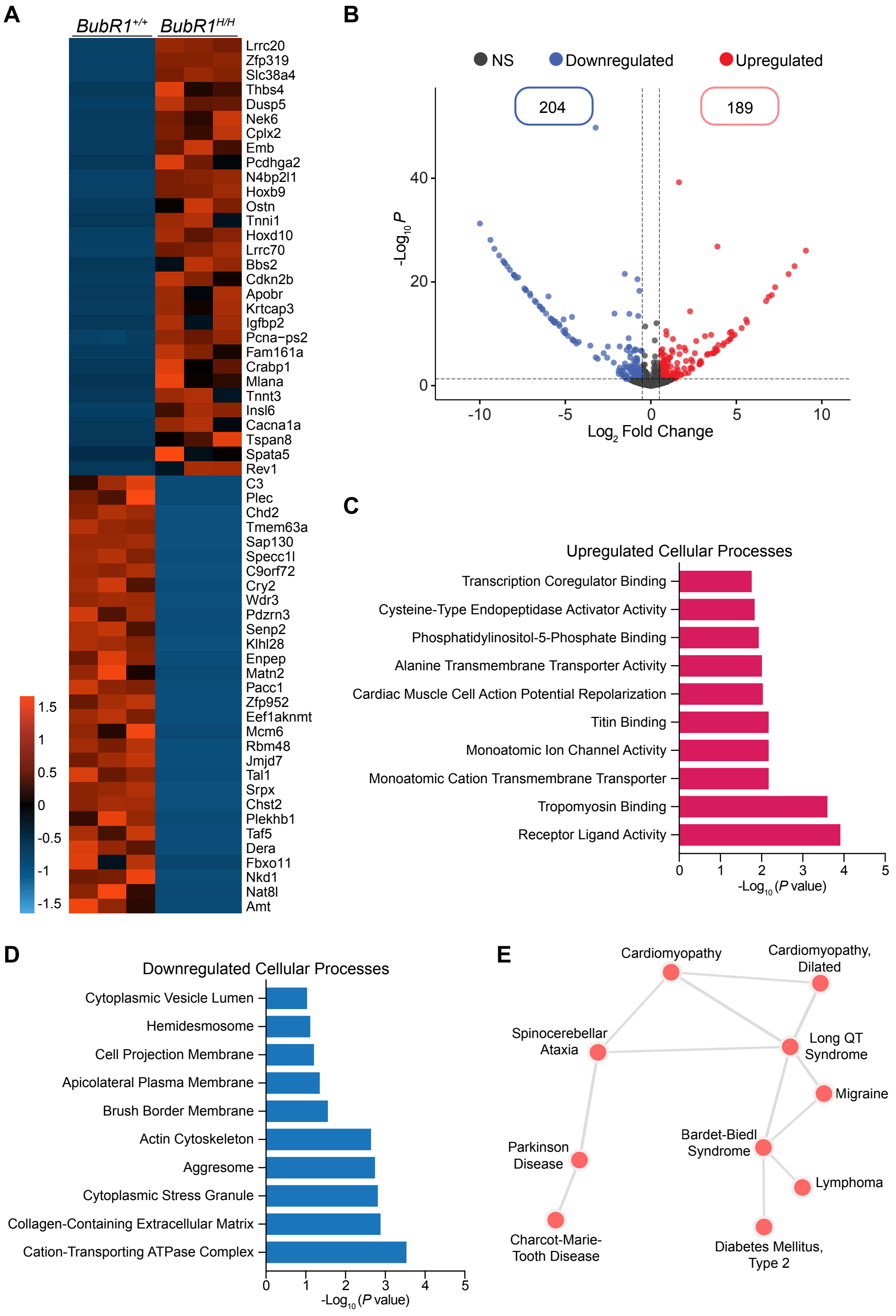
A
BubR1+/+
BubR1H/H
Lrrc20
Zfp319
Slc38a4
Thbs4
Dusp5
Nek6
Cplx2
Emb
Pcdhga2
N4bp2l1
Hoxb9
Ostn
Tnni1
Hoxd10
Lrrc70
Bbs2
Cdkn2b
Apobr
Krtcap3
Igfbp2
Pcna−ps2
Fam161a
Crabp1
Mlana
Tnnt3
Insl6
Cacna1a
Tspan8
Spata5
Rev1
C3
Plec
Chd2
Tmem63a
Sap130
Specc1l
C9orf72
Cry2
Wdr3
Pdzrn3
Senp2
Klhl28
Enpep
Matn2
Pacc1
Zfp952
Eef1aknmt
Mcm6
Rbm48
Jmjd7
Tal1
Srpx
Chst2
Plekhb1
Taf5
Dera
Fbxo11
Nkd1
Nat8l
Amt
1.5
1
0.5
0
-0.5
-1
-1.5
B
NS
Downregulated
Upregulated
204
189
40
20
0
-Log10 P
-10
-5
0
5
10
Log2 Fold Change
C
Upregulated Cellular Processes
Transcription Coregulator Binding
Cysteine-Type Endopeptidase Activator Activity
Phosphatidylinositol-5-Phosphate Binding
Alanine Transmembrane Transporter Activity
Cardiac Muscle Cell Action Potential Repolarization
Titin Binding
Monoatomic Ion Channel Activity
Monoatomic Cation Transmembrane Transporter
Tropomyosin Binding
Receptor Ligand Activity
0
1
2
3
4
5
-Log10 (P value)
D
Downregulated Cellular Processes
Cytoplasmic Vesicle Lumen
Hemidesmosome
Cell Projection Membrane
Apicolateral Plasma Membrane
Brush Border Membrane
Actin Cytoskeleton
Aggresome
Cytoplasmic Stress Granule
Collagen-Containing Extracellular Matrix
Cation-Transporting ATPase Complex
0
1
2
3
4
5
-Log10 (P value)
E
Cardiomyopathy
Cardiomyopathy, Dilated
Spinocerebellar Ataxia
Long QT Syndrome
Migraine
Parkinson Disease
Bardet-Biedl Syndrome
Lymphoma
Charcot-Marie-Tooth Disease
Diabetes Mellitus, Type 2

Figure S3: Global transcriptomic alterations in female BubR1 hypomorphic hearts. A. Heatmap of the top 30 upregulated and downregulated genes in female BubR1 hypomorphic hearts. **B.** Volcano plot showing the number of significantly dysregulated genes using a threshold of $log_2FC > 0.5$ and p value < 0.05. **C.** GO analysis carried out on biological processes of the top upregulated genes in the female BubR1 hypomorphic hearts. **D.** GO analysis carried out on biological processes of the top downregulated genes in the female BubR1 hypomorphic hearts. **E.** Diseased based Enrichr analysis on the top differentially dysregulated genes in the female BubR1 hypomorphic hearts.

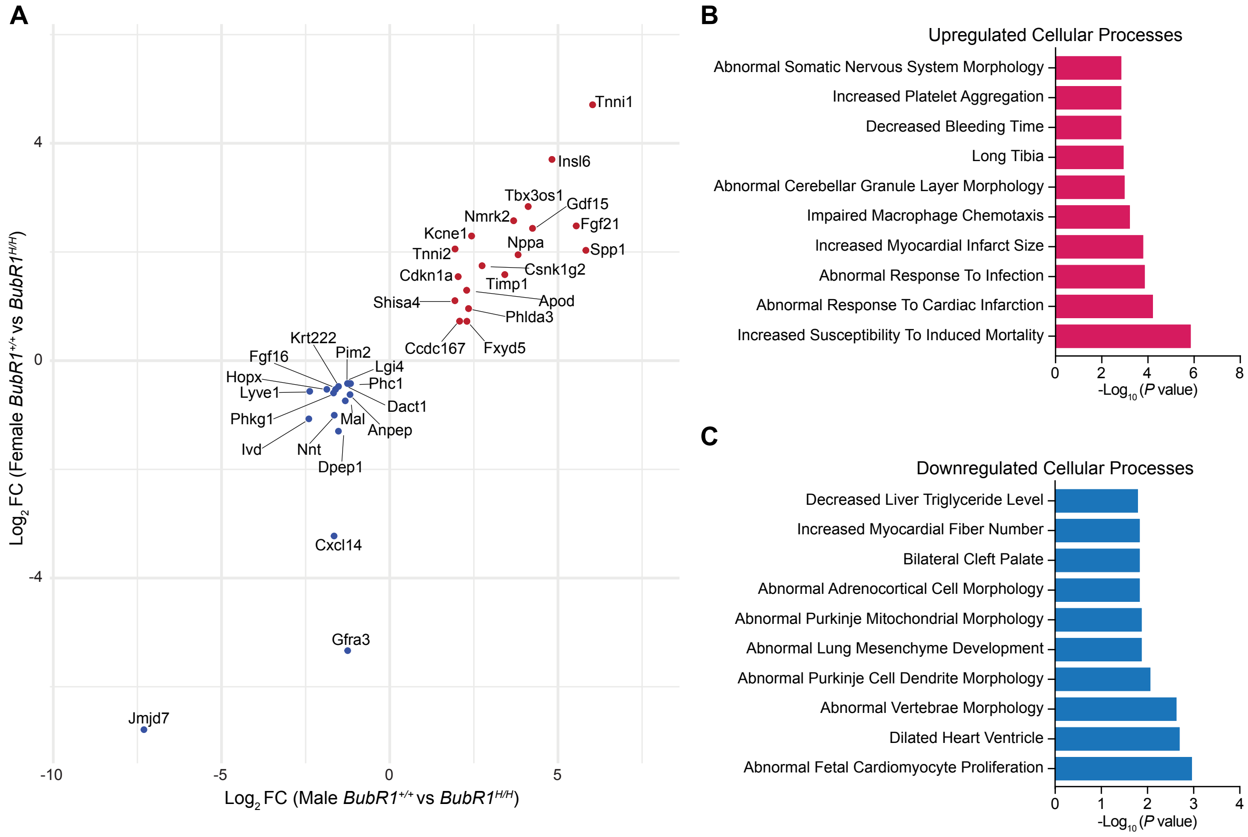

Figure S4: Comparative analysis between male and female transcriptomic alterations. A. Scatterplot showing the top upregulated and downregulated genes between the male and female datasets. **B.** GO analysis carried out on biological processes of the top commonly upregulated genes **C.** GO analysis carried out on biological processes of the top commonly downregulated genes in the male and female BubR1 hypomorphic hearts.

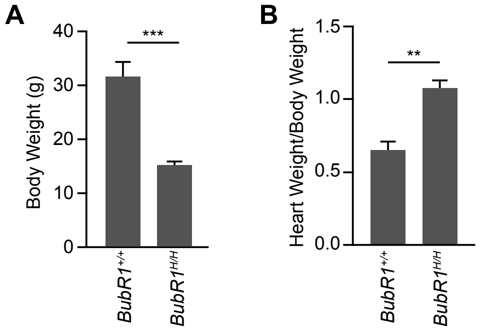

Figure S5: Cardiac hypertrophy analysis of BubR1 hypomorphic hearts. A. Comparison of heart weight between wild-type and BubR1 hypomorphic hearts. **B.** Ratio of heart weight to body weight in wild-type and BubR1 hypomorphic hearts. Statistical significance was calculated by Student's *t*-test. Error bars represent mean ± SEM (n=6). ** $p < 0.01$, *** $p < 0.001$.

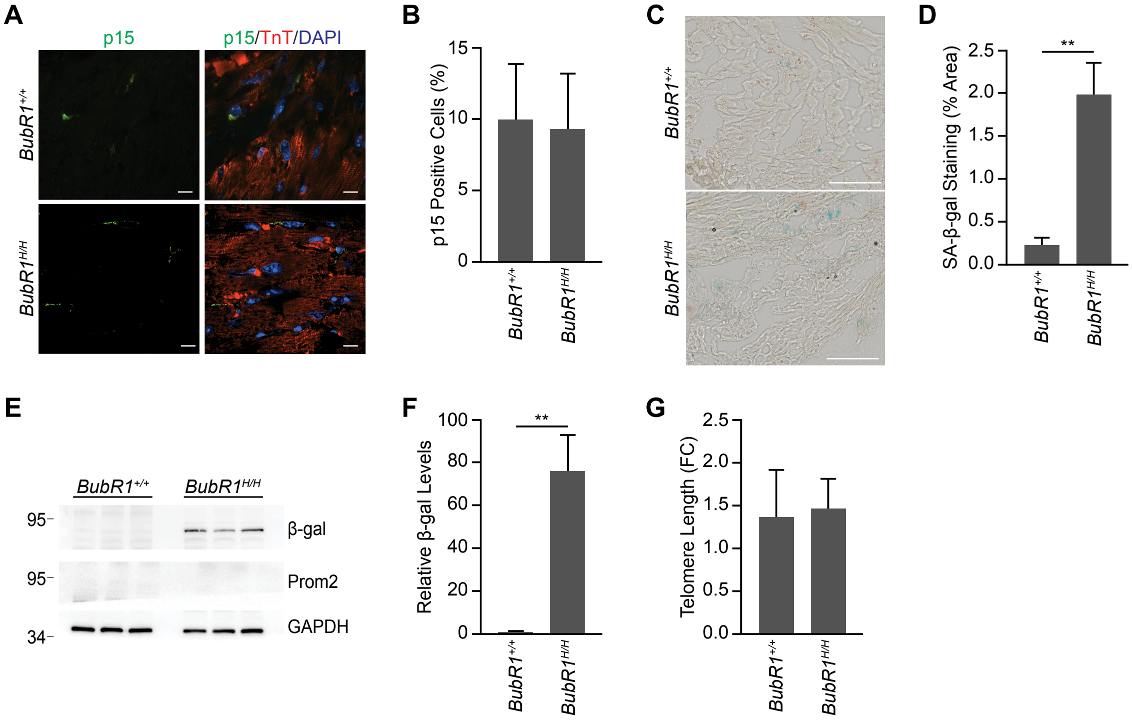

Figure S6: Analysis of senescence markers in BubR1 hypomorphic hearts. A. Confocal microscopy images of p15 positive cells present in wild-type and BubR1 hypomorphic hearts. Scale bar, 10µm. **B.** Quantification of percent of p15 positive cells (n=5). **C.** Representative images of SA-β-gal-stained frozen heart sections. Scale bar, 100µm. **D.** Percent area of SA-β-gal staining quantified through ImageJ software (n=4). **E.** Immunoblot showing the level of β-galactosidase and Prom2 in wild-type versus BubR1 hypomorphic hearts. **F.** Quantification of the relative level of β-gal based on E (n=3). **G.** Telomere length in 4-month-old hearts isolated from wild-type and BubR1 hypomorphic mice (n=7). Statistical significance was calculated by Student's *t*-test. Error bars represent mean ± SEM. ns = not significant. ** $p < 0.01$.

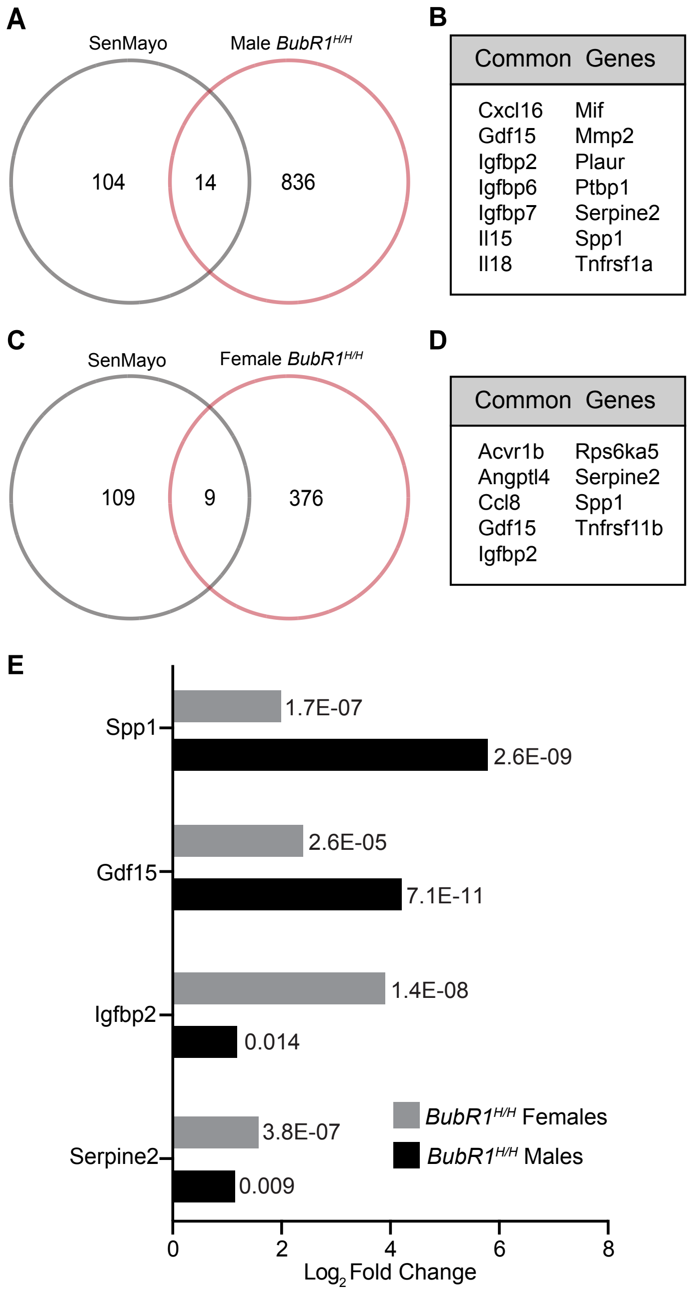

Figure S7: Upregulated expression of SenMayo gene sets in male and female BubR1 hypomorphic hearts. A. Venn diagram showing the overlap between SenMayo genes and those upregulated in male BubR1 hypomorphic hearts. All genes with a p value < 0.05 were included in the Venn diagram analysis. **B.** List of shared genes identified in panel A. **C.** Venn diagram illustrating the overlap between SenMayo genes and those upregulated in female BubR1 hypomorphic hearts. **D.** List of shared genes identified in panel C. **E.** Fold change of common SenMayo genes in male and female hypomorphic hearts relative to their wild-type controls, determined by DESeq analysis. Corresponding p values of the fold changes are indicated on the graph.

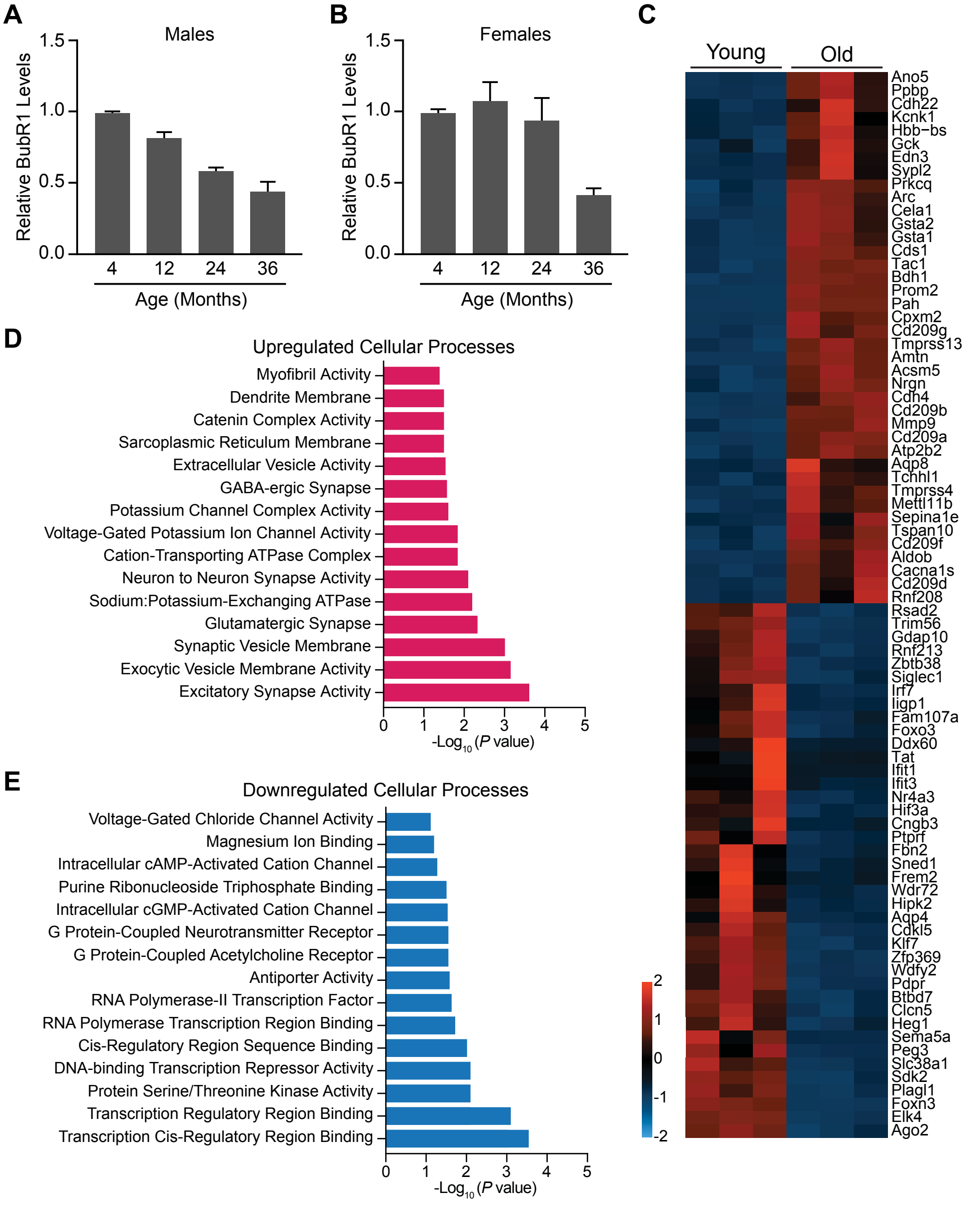

Figure S8: Relative BubR1 levels across different ages and transcriptomic comparison between young and old hearts. A-B. Relative BubR1 levels in male hearts (A) and female hearts (B) (n=2). **C.** Heatmap of the top 30 upregulated and downregulated genes in 22-month-old mouse hearts as compared to 4-month-old hearts. **D.** GO analysis carried out on biological processes of the top upregulated genes in the 22-month-old hearts. **E.** GO analysis carried out on biological processes of the top downregulated genes in the 22-month-old hearts. Error bars represent mean ± SEM.

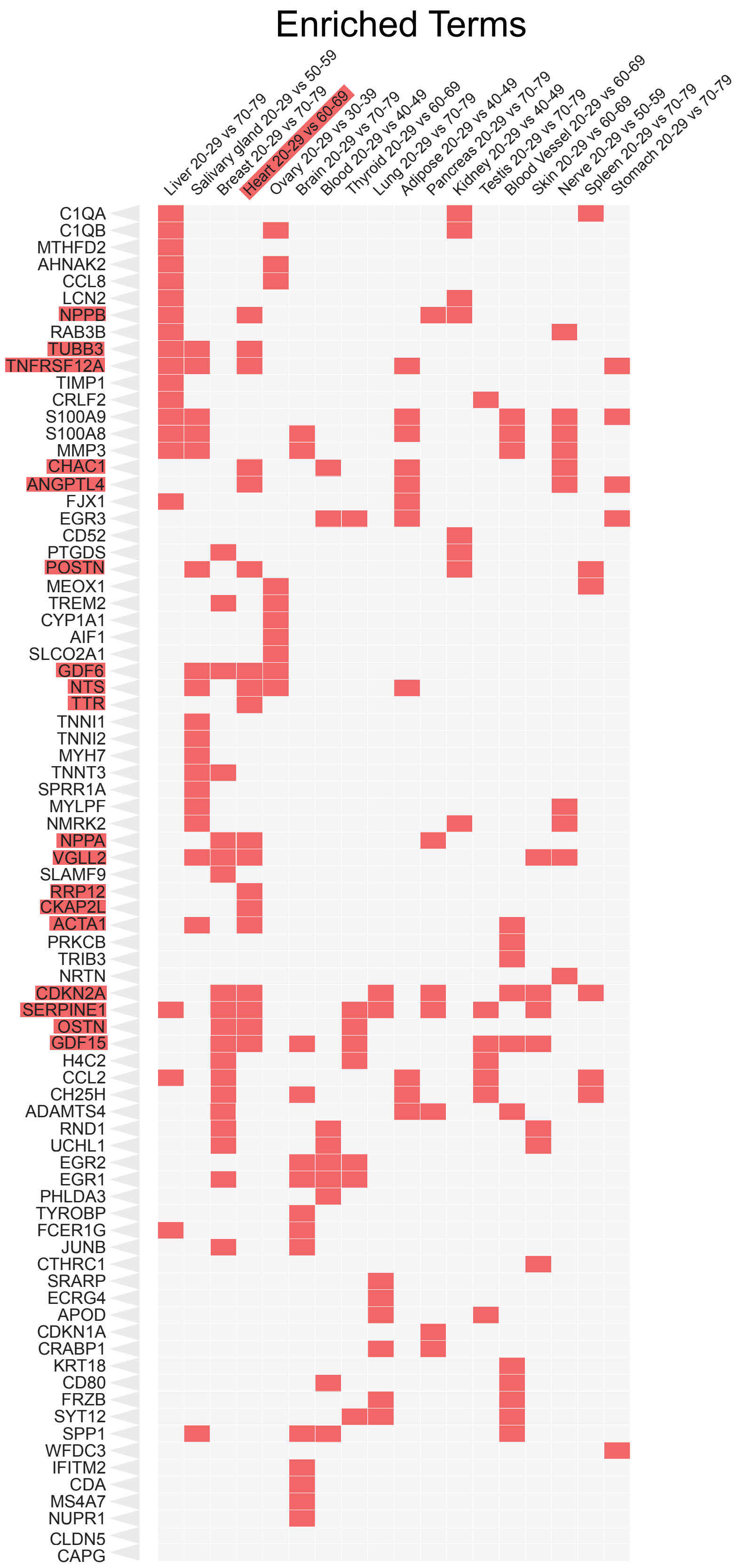
Enriched Terms
Liver 20-29 vs 70-79
Salivary gland 20-29 vs 50-59
Breast 20-29 vs 70-79
Heart 20-29 vs 60-69
Ovary 20-29 vs 30-39
Brain 20-29 vs 70-79
Blood 20-29 vs 40-49
Thyroid 20-29 vs 60-69
Lung 20-29 vs 70-79
Adipose 20-29 vs 40-49
Pancreas 20-29 vs 70-79
Kidney 20-29 vs 40-49
Testis 20-29 vs 70-79
Blood Vessel 20-29 vs 60-69
Skin 20-29 vs 60-69
Nerve 20-29 vs 50-59
Spleen 20-29 vs 70-79
Stomach 20-29 vs 70-79
C1QA
C1QB
MTHFD2
AHNAK2
CCL8
LCN2
NPPB
RAB3B
TUBB3
TNFRSF12A
TIMP1
CRLF2
S100A9
S100A8
MMP3
CHAC1
ANGPTL4
FJX1
EGR3
CD52
PTGDS
POSTN
MEOX1
TREM2
CYP1A1
AIF1
SLCO2A1
GDF6
NTS
TTR
TNNI1
TNNI2
MYH7
TNNT3
SPRR1A
MYLPF
NMRK2
NPPA
VGLL2
SLAMF9
RRP12
CKAP2L
ACTA1
PRKCB
TRIB3
NRTN
CDKN2A
SERPINE1
OSTN
GDF15
H4C2
CCL2
CH25H
ADAMTS4
RND1
UCHL1
EGR2
EGR1
PHLDA3
TYROBP
FCER1G
JUNB
CTHRC1
SRARP
ECRG4
APOD
CDKN1A
CRABP1
KRT18
CD80
FRZB
SYT12
SPP1
WFDC3
IFITM2
CDA
MS4A7
NUPR1
CLDN5
CAPG

Figure S9: GTEx analysis of overexpressed genes in BubR1 hypomorphic hearts. GTEx based analysis of age associated genes in various organs. Genes which are correlated with age in the cardiac system are highlighted.

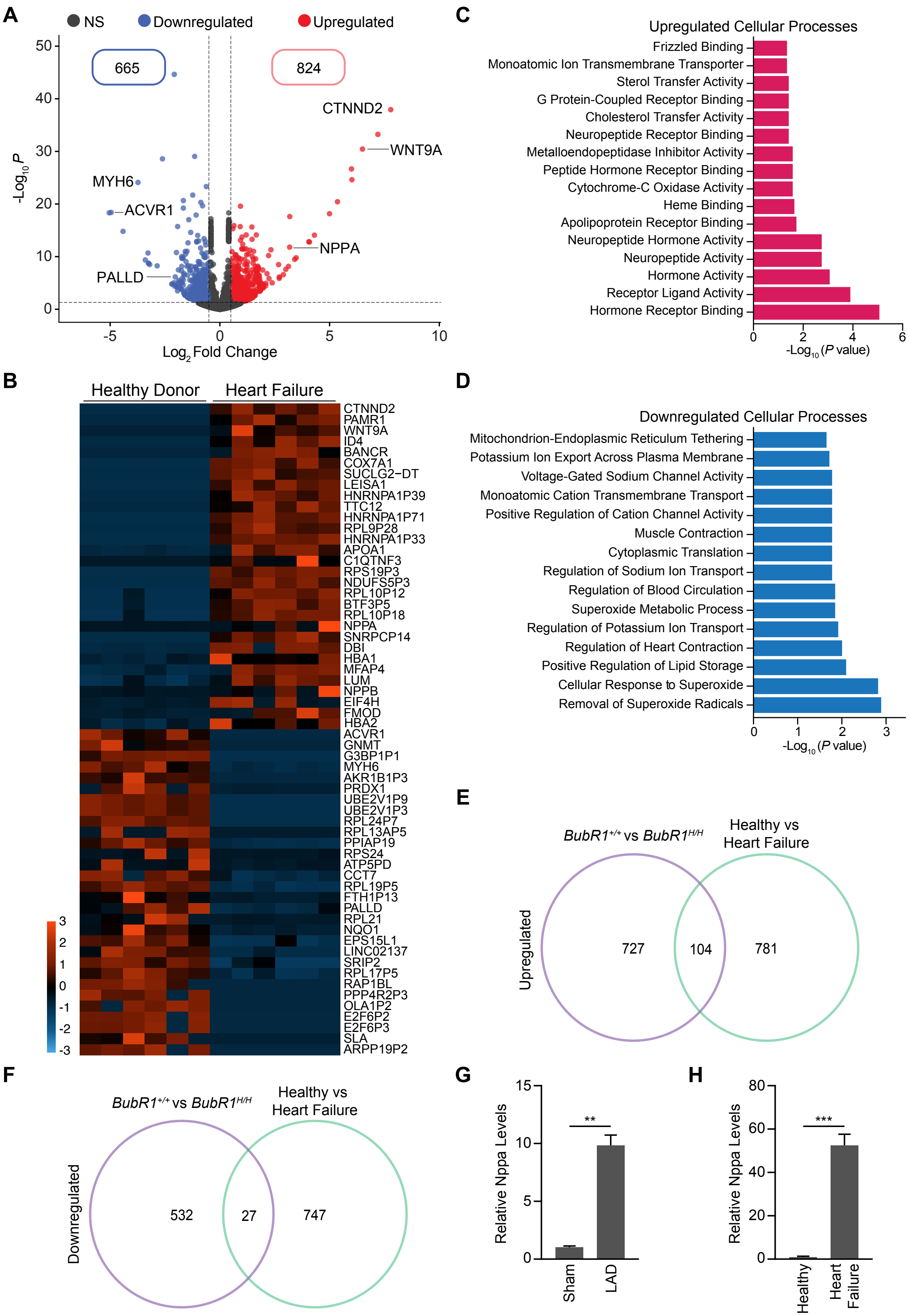

A
NS
Downregulated
Upregulated
665
824
CTNND2
WNT9A
MYH6
ACVR1
NPPA
PALLD
-Log10 P
Log2 Fold Change
50
40
30
20
10
0
-5
0
5
10
B
Healthy Donor
Heart Failure
CTNND2
PAMR1
WNT9A
ID4
BANCR
COX7A1
SUCLG2-DT
LEISA1
HNRNPA1P39
TTC12
HNRNPA1P71
RPL9P28
HNRNPA1P33
APOA1
C1QTNF3
RPS19P3
NDUFS5P3
RPL10P12
BTF3P5
RPL10P18
NPPA
SNRPCP14
DBI
HBA1
MFAP4
LUM
NPPB
EIF4H
FMOD
HBA2
ACVR1
GNMT
G3BP1P1
MYH6
AKR1B1P3
PRDX1
UBE2V1P9
UBE2V1P3
RPL24P7
RPL13AP5
PPIAP19
RPS24
ATP5PD
CCT7
RPL19P5
FTH1P13
PALLD
RPL21
NQO1
EPS15L1
LINC02137
SRIP2
RPL17P5
RAP1BL
PPP4R2P3
OLA1P2
E2F6P2
E2F6P3
SLA
ARPP19P2
3
2
1
0
-1
-2
-3
C
Upregulated Cellular Processes
Frizzled Binding
Monoatomic Ion Transmembrane Transporter
Sterol Transfer Activity
G Protein-Coupled Receptor Binding
Cholesterol Transfer Activity
Neuropeptide Receptor Binding
Metalloendopeptidase Inhibitor Activity
Peptide Hormone Receptor Binding
Cytochrome-C Oxidase Activity
Heme Binding
Apolipoprotein Receptor Binding
Neuropeptide Hormone Activity
Neuropeptide Activity
Hormone Activity
Receptor Ligand Activity
Hormone Receptor Binding
0
2
4
6
-Log10 (P value)
D
Downregulated Cellular Processes
Mitochondrion-Endoplasmic Reticulum Tethering
Potassium Ion Export Across Plasma Membrane
Voltage-Gated Sodium Channel Activity
Monoatomic Cation Transmembrane Transport
Positive Regulation of Cation Channel Activity
Muscle Contraction
Cytoplasmic Translation
Regulation of Sodium Ion Transport
Regulation of Blood Circulation
Superoxide Metabolic Process
Regulation of Potassium Ion Transport
Regulation of Heart Contraction
Positive Regulation of Lipid Storage
Cellular Response to Superoxide
Removal of Superoxide Radicals
0
1
2
3
-Log10 (P value)
E
BubR1+/+ vs BubR1H/H
Healthy vs
Heart Failure
Upregulated
727
104
781
F
BubR1+/+ vs BubR1H/H
Healthy vs
Heart Failure
Downregulated
532
27
747
G
Relative Nppa Levels
15
10
5
0
**
Sham
LAD
H
Relative Nppa Levels
80
60
40
20
0
***
Healthy
Heart
Failure

Figure S10: Transcriptomic analysis of heart failure samples compared to healthy hearts. A. Volcano plot showing the number of significantly dysregulated genes in healthy donor hearts versus heart failure samples using a threshold of $log_2FC > 0.5$ and p value < 0.05. **B.** Heatmap of the top 30 upregulated and downregulated genes. **C.** GO analysis carried out on biological processes of the top upregulated genes in heart failure samples **D.** GO analysis carried out on biological processes of the top downregulated genes in heart failure samples. **E-F.** Venn-diagram showing the number of commonly (E) upregulated genes and (F) downregulated genes between the wild-type versus BubR1 hypomorphic dataset and healthy donor versus heart failure dataset. All genes with a p value < 0.05 were included in the Venn-diagram analysis. **G.** Quantification of Nppa in LAD surgery induced heart failure mice hearts. **H.** Quantification of Nppa in heart failure patients compared to healthy donor hearts. Statistical significance was calculated by Student's *t*-test. Error bars represent mean ± SEM (n=3). ** $p < 0.01$, *** $p < 0.001$.

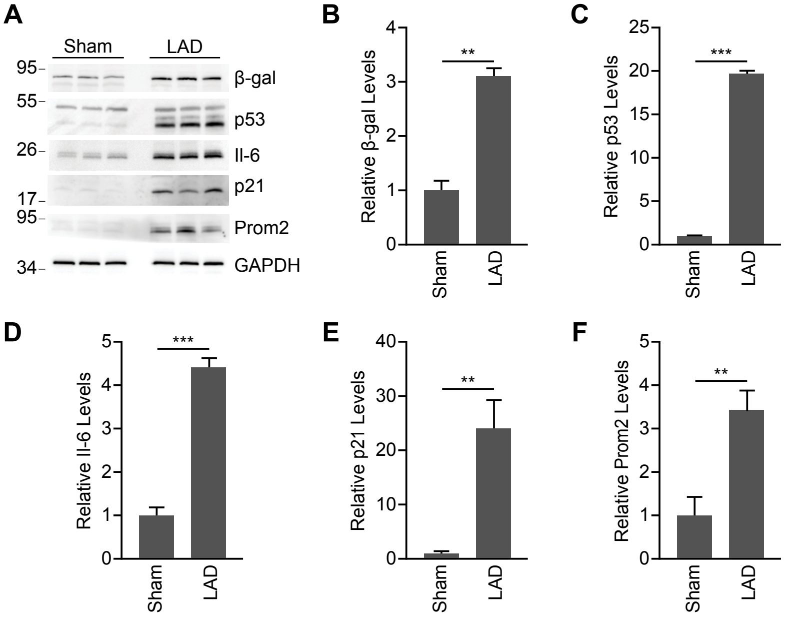

Figure S11: Senescence marker levels in hearts isolated from LAD mouse model. A. Immunoblot showing the levels of β-gal, p53, Il-6, p21, Prom2 in sham versus LAD hearts. **B-F.** Quantification of β-gal (B), p53 (C), Il-6 (D), p21 (E), and Prom2 (F) in LAD hearts. Statistical significance was calculated by Student's *t*-test. Error bars represent mean ± SEM (n=3). ** $p < 0.01$, *** $p < 0.001$.

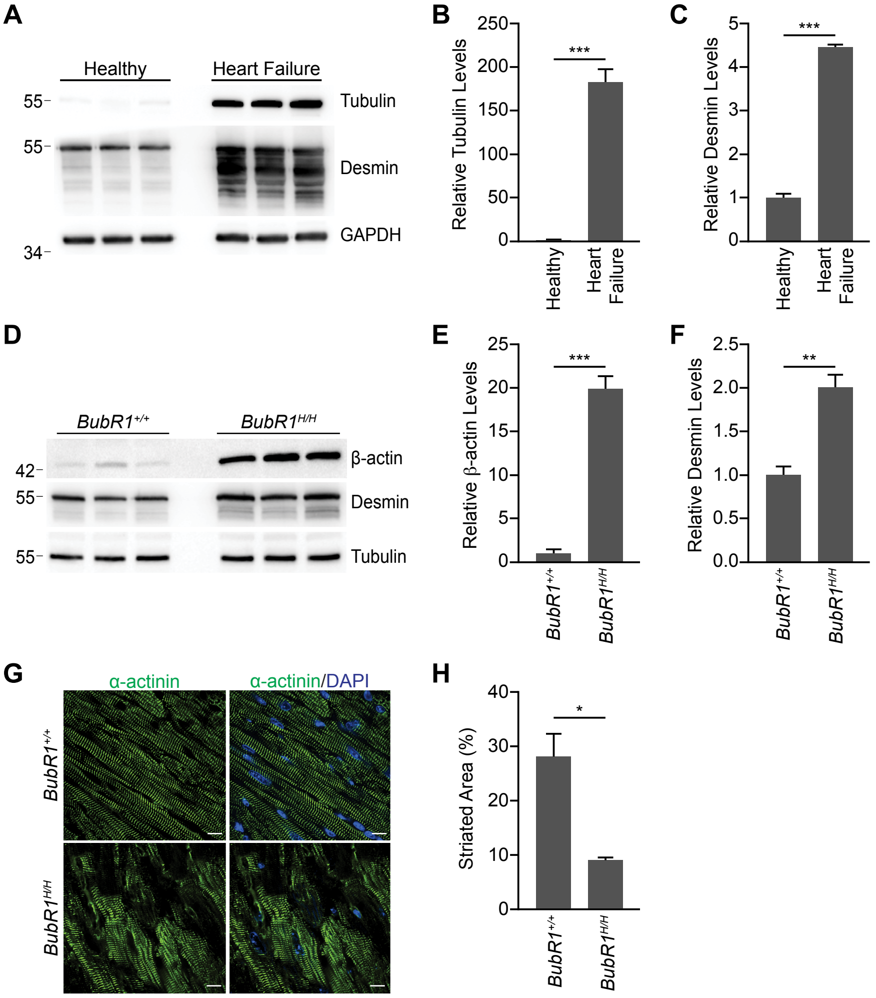

Figure S12: Upregulation of cytoskeletal structural proteins in heart failure patients and BubR1 hypomorphic hearts. A. Immunoblot showing the level of tubulin and desmin in heart failure patients and healthy donor hearts. **B-C.** Quantification of tubulin (B) and desmin (C) in heart failure samples compared to healthy donor hearts. **D.** Immunoblot of β-actin and desmin in wild-type and BubR1 hypomorphic hearts. **E-F.** Quantification of β-actin (E) and desmin (F) in wild-type and BubR1 hypomorphic hearts. **G.** Confocal microscopy images of sarcomere striations visualized with α-actinin present in wild-type and BubR1 hypomorphic hearts. Scale bar, 10μm. **H.** Percent of striated area based on G (n=3). Statistical significance was calculated by Student's *t*-test. Error bars represent mean ± SEM (n=3). * $p < 0.05$, ** $p < 0.01$, *** $p < 0.001$.

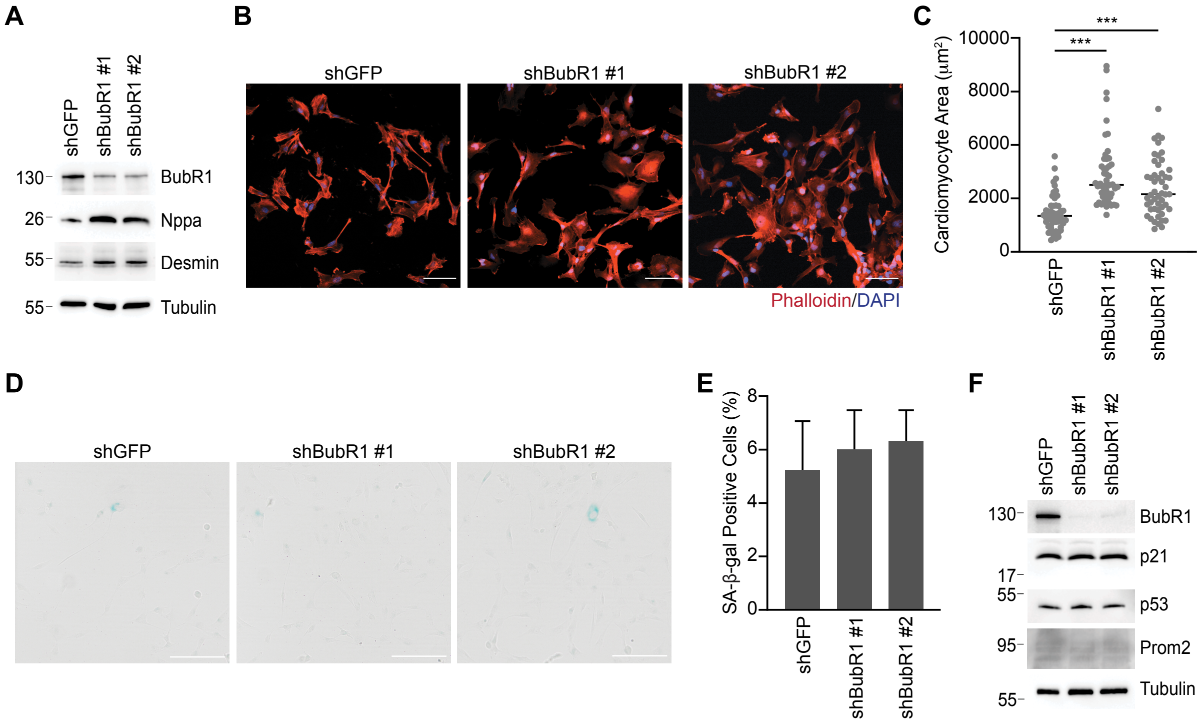

Figure S13: Analysis of cardiac hypertrophy, cytoskeletal remodeling, and senescence markers in BubR1 depleted AC16 cardiomyocytes. A. Immunoblot showing the levels of Nppa and desmin in shGFP (control) and BubR1 shRNA treated AC16 cells. **B.** Representative confocal images of Phalloidin and DAPI stained AC16 cells, Scale bar 50 μm. **C.** Comparison of cardiomyocyte area between shGFP versus and shBubR1 AC16 cells (n=50). **D.** Representative images of SA-β-gal-stained AC16 cells. Scale bar, 200μm. **E.** Quantification of SA-β-gal positive cells (n=3). **F.** Immunoblot analysis showing the levels of p21, p53, and Prom2 in shBubR1 AC16 cells as compared to the control (shGFP) cells. Statistical significance was calculated by Student's *t*-test. Error bars represent mean ± SEM. *** $p < 0.001$, ns = not significant.

Supplementary Table Legends:

Supplementary Table 1: Significantly upregulated and downregulated genes in BubR1 hypomorphic hearts compared to wild-type hearts (Sheet 1). Significantly upregulated and downregulated genes in male BubR1 hypomorphic hearts compared to wild-type hearts (Sheet 2). Significantly upregulated and downregulated genes in female BubR1 hypomorphic hearts compared to wild-type hearts (Sheet 3).

Supplementary Table 2: Significantly upregulated and downregulated genes in young (4-month-old) and aged (22-month-old) hearts (Sheet 1). Common upregulated and downregulated genes between BubR1 hypomorphic hearts and aged mouse hearts (Sheet 2).

Supplementary Table 3: Significantly upregulated and downregulated genes in heart failure patient samples compared to healthy donor hearts (Sheet 1). Common upregulated and downregulated genes between BubR1 hypomorphic hearts and heart failure patient samples (Sheet 2).

Supplementary Table 4: Clinical details of the healthy donor individuals and heart failure patients.

Extended Materials and Methods

DNA isolation and genotyping

BubR1 hypomorphic heterozygotes were bred to obtain wild-type and homozygous hypomorphic mice. P15 pups were genotyped by tail clipping followed by tail lysis for DNA extraction. We incubated the tissues in 600 μL tail lysis buffer (100 mM Tris-HCl, pH 8.0, 10 mM EDTA, 200 mM NaCl, and 1% SDS), supplemented with 60 μg/ml of Proteinase K overnight at 55℃. The samples were incubated in 400 μL chloroform, 0.6 M potassium acetate followed by centrifugation at 14,000 rpm for 20 minutes at 4°C. The aqueous phase was transferred to a new tube with 800 μL of 100% ethanol. The samples were mixed and centrifuged at 14,000 rpm for 20 minutes at 4°C and ethanol was aspirated. The DNA pellet was washed with ice cold 70% ethanol and centrifuged as described above for 5 min. The ethanol was removed, and pellets were dried at 37°C for 10 minutes followed by resuspending in Milli-Q water (Purelab Ultra). Primers used for genotyping were: Forward: 5'GTAAGTCTATTTCTCCTGGATTAAGTAG; Reverse 1: 5'CATCTGTGTACCATACGTGTGTCTGG; Reverse 2: 5' ATATTGCTGAAGAGCTTGGCGGCG. PCR products for wild-type allele was 300 bp and 450 bp for hypomorphic allele. Protocol for the thermocycler (Miniamp Plus Applied Biosystems) was as follows: denaturing at 94°C for 5 minutes, 35 cycles of annealing at 60°C for 90 seconds and extension and at 72°C for 150 seconds, followed by a final extension at 72°C for 5 minutes. PCR products were run on 2.5% agarose gel and images were taken with Bio-Rad ChemiDoc XRS+ molecular imager.

Comparative transcriptomics analysis

Bulk RNA seq data for the analysis of young and aged hearts is available in NCBI GEO under the accession number SRP043967. We used GSM1423595, GSM1423596, and GSM1423597 for younger hearts while GSM1423610, GSM1423611, and GSM1423612 were used for older hearts.

Bulk RNA seq data for the analysis of healthy donor and heart failure patients is available in NCBI GEO under GSE116250. We used healthy donor hearts samples from the following GEO accession numbers: GSM3219571, GSM3219567, GSM3219568, GSM3219558, GSM3219570, and GSM3219565. Heart failure samples belonged to the following GEO accession numbers: GSM3219598, GSM3219587, GSM3219600, GSM3219588, GSM3219594, and GSM3219573.

A list of significantly upregulated and downregulated genes in BubR1 hypomorphic hearts compared to wild-type hearts is provided in Supplementary Table 1. A list of significantly upregulated and downregulated genes in young versus old hearts is provided in Supplementary

Table 2 (sheet 1). A list of common upregulated and downregulated genes between BubR1 hypomorphic hearts and old hearts is provided in Supplementary Table 2 (sheet 2). A list of significantly upregulated and downregulated genes in heart failure patient samples compared to healthy donor hearts is provided in Supplementary Table 3 (sheet 1). A list of common upregulated and downregulated genes between BubR1 hypomorphic hearts and heart failure patient samples is provided in Supplementary Table 3 (sheet 2).

Immunoblotting

Mice were euthanized at 16 weeks of age, and whole hearts were isolated, washed in PBS, snap-frozen in liquid nitrogen, and stored at -80°C. To extract protein, a 10 mg tissue sample from the apex of the heart was excised and homogenized using a glass homogenizer in 500 µL of RIPA lysis buffer (150 mM NaCl, 5 mM EDTA pH 8.0, 50 mM Tris pH 8.0, 1.0% NP-40, 0.5% sodium deoxycholate, and 0.1% SDS) with Halt Protease and Phosphatase Inhibitors (Thermo Scientific, 78445). Total protein concentration was quantified using the DC Protein Assay Kit (Bio-Rad), and Laemmli buffer was added to achieve a 1X concentration. Proteins were boiled at 95°C for 10 minutes and separated on a 10% or 15% SDS-PAGE gel. The resolved proteins were transferred to a nitrocellulose membrane and blocked with 5% dry milk diluted in Tris-Buffered Saline Tween-20 (TBS-T), followed by overnight incubation with primary antibodies at 4°C. Membranes were washed three times with TBS-T and incubated with secondary antibodies for 45 minutes at room temperature. After extensive washing, ECL substrate (Millipore, WBULP) was applied according to the manufacturer's instructions, and detection was performed using the Bio-Rad ChemiDoc XRS+ molecular imager.

Primary antibodies used for immunoblotting included alpha-tubulin (Santa Cruz Biotechnology, sc-23948), p21 (Cell Signaling Technology, 64016), p53 (Cell Signaling Technology, 2524), IL-6 (Cell Signaling Technology, 12912), αSMA (eBioscience, 2604946), Nppa (Proteintech, 27426-1-AP), desmin (Proteintech, CL594-16520), GAPDH (Cell Signaling Technology, 14C10), Prom2 (Biotechne, MAB2024), β-Galactosidase (Cell Signaling Technology, 27198), and β-actin (Cell Signaling Technology, 4970S). An antibody directed against BubR1 amino acids 1-350 was custom generated (Yenzyme). Secondary antibodies included anti-mouse HRP conjugated antibody (RD Systems, HAF018) and anti-rabbit IgG, HRP-linked antibody (Cell Signaling Technology, 7074).

Generation of BubR1 Knockdown AC16 Cells

Vector plasmids (pTRIPZ) containing shRNA sequence against human BUB1B were purchased from Horizon. Clone ID for shBubR1 #1 was V3THS_359184 and the shRNA sequence was ACATCTAGATCTTCTTCCG. Clone ID for shBubR1 #2 was V3THS_359186 and the shRNA sequence was AGGCTTAGACAACTCTGCT. Control vector contained shRNA against GFP. To produce viral shRNA containing viral particles, HEK 293T cells were seeded and transfected with 5 μg of the pTRIPZ lentiviral transfer vector, 3.5 μg of the packaging vector (psPAX2), and 1.5 μg of the envelope vector (pMD2.G) using the calcium phosphate transfection method. Virus containing media was harvested at 48 hours post transfection and filtered through 0.45 μM PES membrane. AC16 cells were transduced with virus containing media (diluted 1:1 with fresh media) in the presence of 10 μg/ml polybrene (Santa cruz technology, sc-134220). Puromycin (Thermo scientific, J67236.XF) selection was initiated 48 hours after infection and terminated once uninfected control cells died. To induce the shRNA, Doxycycline (1μg/ml) was added to the cells for at least 36 hours before collecting protein lysates. Western blot analysis was utilized to confirm BubR1 knockdown.

Immunoblotting analysis of cultured cells

AC16 cells were cultured in DME/F-12 (1:1) (Cytvia, SH30023.01) supplemented with 10% Fetal Bovine Serum (FBS) (Cytvia, SH30910.03), 1% penicillin-streptomycin (Gibco, 15140-122) and incubated at 37°C in a humidified atmosphere with 5% CO_2. Cells were treated with 1μg/ml Doxycycline (Thermo scientific, 446060250) for 36 hours followed by lysis in IPLS buffer (50 mM Tris-HCl, 0.5 mM EDTA, 150 mM NaCl, 0.5% NP-40, 1X HALT protease and phosphatase inhibitor cocktail) for 30 minutes on ice with agitation. Lysates were centrifuged at 13,000 rpm at 4°C for 5 min. The protein concentration was quantified with DC Protein Assay Kit. Protein concentrations were normalized and Laemmli buffer added to 1X concentration. Proteins were boiled for 10 minutes at 95°C before separation by SDS-PAGE on a 10% gel. The resolved gels were transferred to a nitrocellulose membrane, and the membranes were blocked with 5% blocking milk diluted in 1X TBS with 0.1% Tween-20, followed by incubation with primary antibody overnight at 4°C, washed, and subsequently incubated with anti-rabbit or anti-mouse HRP conjugated secondary antibodies for 45 minutes at room temperature. Membranes were washed extensively followed by addition of ECL substrate according to the manufacturer's recommendations and detection on Bio-Rad ChemiDoc XRS+ molecular imager.

Primary antibodies included BubR1(BD transduction, 612503), Nppa (Proteintech, 27426-1-AP), desmin (Proteintech, CL594-16520), Tubulin (Sigma Aldrich, T5168), p21 (Santa Cruz

Biotechnology, sc-471), p53 (Cell Signaling Technology, 2524), and Prom2 (Biotechne, MAB2024). Secondary antibodies included anti-mouse HRP conjugated antibody (RD Systems, HAF018) and anti-rabbit IgG, HRP-linked antibody (Cell Signaling Technology, 7074).

Hypertrophy analysis

AC16 cells were seeded in 8-well chamber slides (Lab-Tek II Chamber Slide System, 154941). Cells were treated with 1μg/ml doxycycline for 36 hours to induce shRNA mediated knockdown of BubR1. Cells were rinsed with PBS and fixed with 4% Paraformaldehyde (Chemcruz, sc-281692) for 15 minutes at room temperature followed by three washes with PBS. Alexa Fluor-555 Phalloidin (Cell Signaling Technology, 8953), diluted 1:100 μl in 0.3% BSA in PBS, was added to the cells and incubated for 15 minutes at room temperature followed by a rinse with PBS. Slides were then mounted with mounting medium with DAPI. Images were taken with Nikon Ti-E inverted microscope with a Yokagawa Spinning Disc. Cell area was quantified by NIS-elements AR analysis software using the Bezier tool.

FFPE tissue preparation

Whole hearts were placed in cold PBS immediately following dissection and were washed with PBS to remove blood. Hearts were then fixed in formalin overnight at room temperature followed by 70% ethanol wash. Formalin fixed paraffin embedded tissue (FFPE) sections were prepared with Thermo Scientific excelsior ES tissue processor in which the heart tissues were serially dehydrated in 70%, 95%, and 100% ethanol followed by three washes in xylene and wax. The FFPE blocks were cut on Leica RM 2135 rotary microtome and 8μM thick sections made.

Immunofluorescence on tissue sections

Tissue sections were deparaffinized with two washes in xylene for 5 minutes followed by rehydration by serial washes twice for five minutes each with 100% ethanol, 95% ethanol, 70% ethanol, 50% ethanol. Rehydration was completed in Milli-Q water for 5 minutes followed by heat induced epitope retrieval (HIER) with Tris-EDTA buffer (10 mM Tris Base, 1 mM EDTA, 0.05% Tween 20, pH 9.0). The slides were boiled in Tris-EDTA buffer in a steamer for 30 minutes followed by a cool-down phase at room temperature for 20 minutes. Tissue sections were blocked with 3% bovine serum albumin (Fisher Bioreagents, BP1605) in PBS for 30 minutes at room temperature. Primary antibodies, diluted in 1% bovine serum albumin in PBS, were added and incubated overnight at 4ºC in a humidified chamber. Slides were washed with TBS-T solution for 10 minutes three times followed by incubation with secondary antibody which was diluted in 1%

bovine serum albumin in PBS at room temperature for 1 hour in a humidified chamber protected from light. Slides were then washed three times with TBS-T for 10 minutes, followed by mounting the slides with mounting medium containing DAPI and overlayed with cover slips.

Primary antibodies used for immunofluorescence on tissue sections include p16 (Cell Signaling Technology, 29271), p15 (Cell Signaling Technology, 36303), phosphorylated-H2AX-S139 (Cell Signaling Technology, 2577), α-actinin (Cell Signaling Technology, 6487T), and S100a8 (Proteintech, 15792-1-AP). Secondary antibodies included donkey anti-rabbit Alexa Fluor 488 Molecular Probes (Thermo Fisher Scientific, A-21206), and donkey anti-mouse Alexa Fluor 568 (Thermo Fisher Scientific, A10037).

Senescence β-Galactosidase staining

Senescence β-Galactosidase Staining Kit (Cell Signaling Technology, 9860) was used according to the manufacturer's instructions. Fresh heart tissues were embedded in OCT compound (SAKURA, 4583), frozen, and sectioned at 10 µm thickness with a Leica RM 2135 microtome. SA-β-gal staining was performed at 37°C for 24 hours. AC16 cells were cultured in 8-well chambered slides and treated with 1 µg/ml doxycycline for 36 hours to induce shRNA-mediated depletion of BubR1. Senescence was then assessed following doxycycline treatment as described above for frozen heart sections. Images were acquired using bright-field microscopy (Olympus VS120 slide scanner) and analyzed with Olympus VS-ASW software. Image quantification was performed in ImageJ (National Institutes of Health). For tissue sections, the SA-β-gal-stained area was measured and normalized to the total image area to calculate the percentage of SA-β-gal staining. For AC16 cells, the ratio of SA-β-gal-positive cells to the total cell count was determined.

WGA staining

Tissue sections were deparaffinized with two washes in xylene for 5 minutes followed by rehydration by serial washes twice for five minutes each with 100% ethanol, 95% ethanol, 70% ethanol, 50% ethanol. Rehydration was completed in Milli-Q water for 5 minutes. A hydrophobic barrier was drawn around individual heart sections with a pap pen. For staining with WGA, a 10 µg/ml WGA stain was added to the tissue sections and incubation was carried out for 20 minutes at room temperature in the dark (WGA Invitrogen, W11261). The WGA stain was removed, and tissue sections were washed with PBS followed by mounting the slides with mounting medium containing DAPI (Sigma-Aldrich, F6057) and overlaying with cover slips. Confocal images were acquired with Nikon Ti2 spinning disk confocal and analyzed with NIS elements AR analysis

5.02.01 64-bit software. Total area of individual cell was measured with the Bezier tool and quantified as the area of the ROI.

Masson's trichrome staining

FFPE sections were deparaffinized and rehydrated as described above. We utilized the Trichrome Stain kit (Abcam, ab150686) following the manufacturer's suggested protocol for visualization of collagen deposition in the hearts. ImageJ was used to quantify the area of collagen deposits which were normalized against the entire area of the tissue section and defined as percent fibrosis.

Telomere length quantification

DNA from frozen 4-month-old wild-type and BubR1 hypomorphic hearts were isolated using the Monarch Spin gDNA Extraction Kit (New England Biolabs, T3050) according to the manufacturer's protocol. Telomere length was evaluated by quantitative PCR with reverse transcription (RT-qPCR) with the relative mouse telomere length quantification qPCR assay kit (ScienCell, M8908) according to the manufacturer's protocol.

Striated area quantification

To assess sarcomere organization, α-actinin immunofluorescence was visualized using confocal microscopy. For each biological replicate, three random images were acquired, and the percentage of striated area was quantified. Images were processed in ImageJ (NIH) after converting the image to 8 bit and thresholding to highlight the α-actinin striations. The percentage of striated area was calculated using the formula: (Striated area/total ROI area) ×100. For each biological replicate, the mean percent striation was computed by averaging the values from three independent fields.
